# Clinical characteristics and outcomes of COVID-19 patients with preexisting dementia: a large multicenter propensity-matched Brazilian cohort study

**DOI:** 10.1186/s12877-023-04494-w

**Published:** 2024-01-05

**Authors:** Maria Aparecida Camargos Bicalho, Márlon Juliano Romero Aliberti, Polianna Delfino-Pereira, Victor Schulthais Chagas, Patryk Marques da Silva Rosa, Magda Carvalho Pires, Lucas Emanuel Ferreira Ramos, Adriana Falangola Benjamin Bezerra, Ana Beatriz de Castro Feres, Angélica Gomides dos Reis Gomes, Angelinda Rezende Bhering, Bruno Porto Pessoa, Carla Thais Cândida Alves da Silva, Christiane Corrêa Rodrigues Cimini, Claudia Kimie Suemoto, Cristiana Andrade Coelho Dias, Daniela dos Reis Carazai, Daniela Ponce, Danyelle Romana Alves Rios, Euler Manenti, Fernando Anschau, Joanna d‘Arc Lyra Batista, Joice Coutinho de Alvarenga, Julia Avancini Viguini, Julia Mariot Zanellato, Juliana Machado Rugolo, Karen Brasil Ruschel, Leticia do Nascimento, Luanna Silva Monteiro Menezes, Lucas Moyses Carvalho de Oliveira, Luís César de Castro, Luiz Antônio Nasi, Marcelo Carneiro, Maria Angélica Pires Ferreira, Mariana Frizzo de Godoy, Milton Henriques Guimarães-Júnior, Neimy Ramos de Oliveira, Patricia Klarmann Ziegelmann, Paula Fonseca Porto, Paulo Mascarenhas Mendes, Pedro Gibson Paraíso, Priscilla Pereira dos Reis, Saionara Cristina Francisco, Silvia Ferreira Araújo, Thiago Junqueira Avelino-Silva, Milena Soriano Marcolino

**Affiliations:** 1 Medical School, Universidade Federal de Minas Gerais, Av. Professor Alfredo Balena, 190, Sala 246, Santa Efigênia, Belo Horizonte, Brazil; 2 Cologne University, Albertus-Magnus-Platz, Cologne, 50923 Allemagne; 3 Laboratório de Investigação Médica Em Envelhecimento (LIM-66), Serviço de Geriatria, Hospital das Clínicas HCFMUSP, Faculdade de Medicina, Universidade de São Paulo, São Paulo, Brazil; 4 Research Institute, Hospital Sírio-Libanês, São Paulo, Brazil; 5 Institute for Health Technology Assessment (IATS), R. Ramiro Barcelos, 2359, Porto Alegre, Brazil; 6 Medical School, Universidade Federal de Viçosa, Av. Peter Henry Rolfs, S/N, Viçosa, Brazil; 7 Telehealth Center, University Hospital, Universidade Federal de Minas Gerais, Av. Professor Alfredo Balena, 110, Belo Horizonte, Brazil; 8 Medical School, Centro Universitário de Belo Horizonte, Av. Professor Mário Werneck, 1685, Belo Horizonte, Brazil; 9 Department of Statistics, Universidade Federal de Minas Gerais, Av. Presidente Antônio Carlos, 6627, Belo Horizonte, Brazil; 10 Hospital das Clínicas da Universidade Federal de Pernambuco, Av. Prof. Moraes Rego, 1235, Recife, Brazil; 11 Medical School, Faculdade Ciências Médicas de Minas Gerais, Al. Ezequiel Dias, 275, Belo Horizonte, Brazil; 12 Hospitais da Rede Mater Dei, Av. do Contorno, 9000, Belo Horizonte, Brazil; 13 Hospital UNIMED BH, Av. do Contorno, 3097, Belo Horizonte, Brazil; 14 Hospital Júlia Kubitschek, Av. Professor Alfredo Balena, 190, Belo Horizonte, Brazil; 15 Hospital Santo Antônio, Pç. Dr. Márcio Carvalho Lopes Filho, 501, Curvelo, Brazil; 16 Hospital Santa Rosália, R. Do Cruzeiro, 01, Teófilo Otoni, Brazil; 17 Mucuri Medical School, Universidade Federal dos Vales do Jequitinhonha e Mucuri, R. Cruzeiro, 01, Teófilo Otoni, Brazil; 18 Hospital Nossa Senhora da Conceição and Hospital Cristo Redentor, Av. Francisco Trein, 326, Porto Alegre, Brazil; 19 Botucatu Medical School, Universidade Estadual Paulista “Júlio de Mesquita Filho”, Av. Prof. Mário Rubens Guimarães Montenegro, Botucatu, Brazil; 20 Universidade Federal de São João del-Rei, R. Sebastião Gonçalves Coelho, 400, Divinópolis, Brazil; 21 Hospital Mãe de Deus, R. José de Alencar, 286, Porto Alegre, Brazil; 22 Universidade Federal da Fronteira Sul, Rod. SC-459, Km 02, Fronteira Sul, Chapecó, Brazil; 23 Hospital Regional do Oeste, Hospital Regional do Oeste, Florianópolis street, Brazil; 24 Hospital João XXIII, Av. Professor Alfredo Balena, 400, Belo Horizonte, Brazil; 25 Hospital SOS Cárdio, Rod. SC-401, 121, Florianópolis, Brazil; 26 Hospital Universitário Canoas, Av. Farroupilha, 8001, Canoas, Brazil; 27 Hospital Universitário de Santa Maria, Av. Roraima, 1000, Santa Maria, Brazil; 28 Hospital Metropolitano Odilon Behrens, R. Formiga, 50, Belo Horizonte, Brazil; 29 Hospital Luxemburgo, R. Gentios, 1350, Belo Horizonte, Brazil; 30 Hospital Universitário Ciências Médicas, R. dos Aimorés, 2896, Belo Horizonte, Brazil; 31 Hospital Bruno Born, Av. Benjamin Constant, 881, Lajeado, Brazil; 32 Hospital Moinhos de Vento, R. Ramiro Barcelos, 910, Porto Alegre, Brazil; 33 Hospital Santa Cruz, R. Fernando Abott, 174, Santa Cruz Do Sul, Brazil; 34 Hospital de Clínicas de Porto Alegre, R. Ramiro Barcelos, 2350, Porto Alegre, Brazil; 35 Hospital São Lucas da PUCRS, Av. Ipiranga, 6690, Porto Alegre, Brazil; 36 Hospital Márcio Cunha, Av. Kiyoshi Tsunawaki, 48, Ipatinga, Brazil; 37 Hospital Eduardo de Menezes, R. Dr. Cristiano Rezende, 2213, Belo Horizonte, Brazil; 38 Hospital Tacchini, R. Dr. José Mário Mônaco, 358, Bento Gonçalves, Brazil; 39 Orizonti - Instituto Oncomed de Saúde e Longevidade, Av. José Do Patrocínio Pontes, 1355, Belo Horizonte, Brazil; 40 Hospital Metropolitano Doutor Célio de Castro, R. Dona Luiza, 311, Belo Horizonte, Brazil; 41 Hospital Semper, Al. Ezequiel Dias, 389, Belo Horizonte, Brazil; 42 Faculdade Israelita de Ciências da Saúde Albert Einstein, Hospital Israelita Albert Einstein, São Paulo, Brazil; 43 Global Brain Health Institute, University of California, Av, Raja Gabaglia, San Francisco, Estoril USA

**Keywords:** COVID-19, Dementia, Hospitalisation, Retrospective study, Multicentre study, Prognosis, Severity, Hospital mortality, Brazil

## Abstract

**Background:**

Although dementia has emerged as an important risk factor for severe SARS-CoV-2 infection, results on COVID-19-related complications and mortality are not consistent. We examined the clinical presentations and outcomes of COVID-19 in a multicentre cohort of in-hospital patients, comparing those with and without dementia.

**Methods:**

This retrospective observational study comprises COVID-19 laboratory-confirmed patients aged ≥ 60 years admitted to 38 hospitals from 19 cities in Brazil. Data were obtained from electronic hospital records. A propensity score analysis was used to match patients with and without dementia (up to 3:1) according to age, sex, comorbidities, year, and hospital of admission. Our primary outcome was in-hospital mortality. We also assessed admission to the intensive care unit (ICU), invasive mechanical ventilation (IMV), kidney replacement therapy (KRT), sepsis, nosocomial infection, and thromboembolic events.

**Results:**

Among 1,556 patients included in the study, 405 (4.5%) had a diagnosis of dementia and 1,151 were matched controls. When compared to matched controls, patients with dementia had a lower frequency of dyspnoea, cough, myalgia, headache, ageusia, and anosmia; and higher frequency of fever and delirium. They also had a lower frequency of ICU admission (32.7% vs. 47.1%, *p* < 0.001) and shorter ICU length of stay (7 vs. 9 days, *p* < 0.026), and a lower frequency of sepsis (17% vs. 24%, *p* = 0.005), KRT (6.4% vs. 13%, *p* < 0.001), and IVM (4.6% vs. 9.8%, *p* = 0.002). There were no differences in hospital mortality between groups.

**Conclusion:**

Clinical manifestations of COVID-19 differ between older inpatients with and without dementia. We observed that dementia alone could not explain the higher short-term mortality following severe COVID-19. Therefore, clinicians should consider other risk factors such as acute morbidity severity and baseline frailty when evaluating the prognosis of older adults with dementia hospitalised with COVID-19.

**Supplementary Information:**

The online version contains supplementary material available at 10.1186/s12877-023-04494-w.

## Background

Currently, more than 55 million people worldwide live with dementia, with nearly 10 million new cases reported every year [1]. Dementia is the seventh leading cause of death among all diseases and one of the major causes of disability among older adults globally. This condition carries significant psychological, physical, economic, and social impacts, not only for people living with dementia, but also for their relatives, carers, and general society [1]. Previous research has highlighted that the Coronavirus Disease 2019 (COVID-19) pandemic could cause more deleterious effects on people living with dementia [2]. These patients are at a higher risk of experiencing severe COVID-19 due to factors such as older age, frailty, inflammation, and the presence of comorbidities, especially cardiovascular diseases [3].

Although several studies have described that all-cause dementia increases the risk for severe COVID-19 [4–8], results regarding complications and mortality related to COVID-19 hospitalisation are not consistent [9, 10]. In fact, disparities across studies could be explained by differences in sociodemographic factors and clinical characteristics of study participants as most studies have not considered such confounders [11, 12]. Moreover, as the pandemic advances and therapeutic options are developed (e.g., vaccines, drug therapies), older individuals, particularly those living with dementia, who are vulnerable to the severe forms of COVID-19, tend to comprise the majority of patients who require acute care for the infection [13]. In this context, we need to understand the particularities of Severe Acute Respiratory Syndrome Coronavirus 2 (SARS-CoV-2) infection among older patients with dementia admitted to hospital.

Therefore, our aim was to investigate the clinical characteristics and outcomes of older patients with dementia hospitalised for COVID-19, comparing their findings with a matched sample of older patients without dementia. We included data from a multicentre cohort comprising 38 hospitals located in different regions of Brazil, a country severely impacted by the COVID-19 pandemic.

## Patients and methods

This study was approved by the National Commission for Research Ethics from the Brazilian Ministry of Health (CAAE 30350820.5.1001.0008). This manuscript adheres to the Strengthening the Reporting of Observational Studies in Epidemiology (STROBE) guidelines [14].

### Study design and patient population

Data from two cohort studies, the Brazilian COVID-19 Registry [15] and the COVID-19 and Frailty (CO-FRAIL) Study [13] were combined and assembled in a multicentre COVID-19 cohort, totaling 38 different hospitals in 19 Brazilian cities. Hospitals were invited to participate in the Brazilian COVID-19 Registry, through social media, radio, and the National Institute of Science and Technology for Health Technology Assessment (*Instituto de Avaliação de Tecnologias em Saúde* – IATS) website, as previously described [15].

**Fig. 1:**
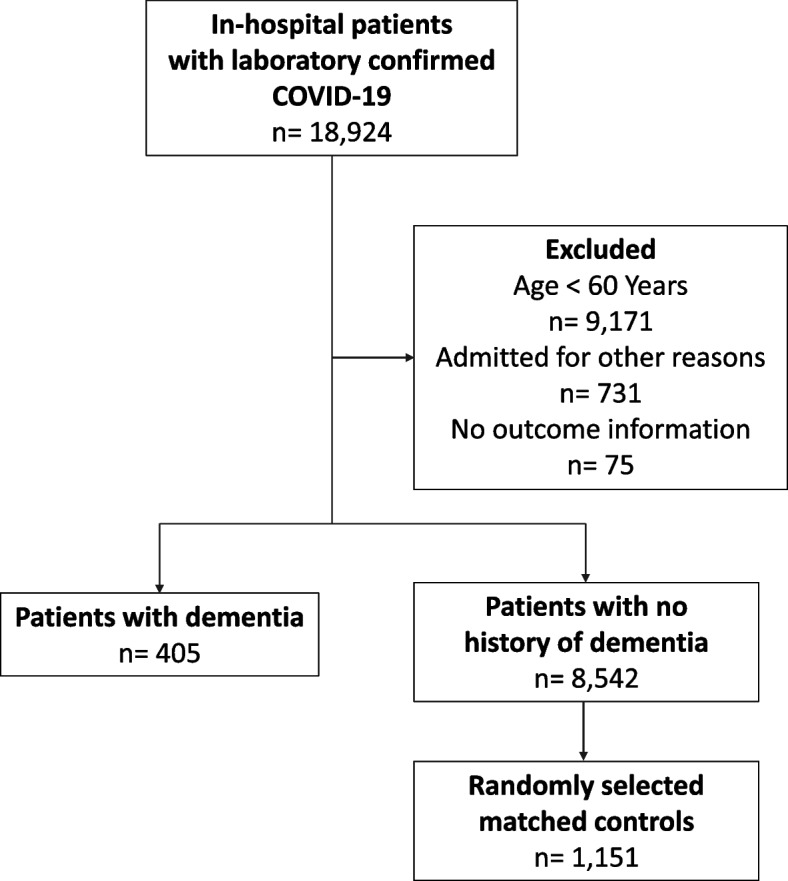
Flowchart of COVID-19 patients included in the study cohort

We included consecutive older adults (≥ 60 years old) with laboratory-confirmed COVID-19 (following the World Health Organization guidance) [16], who were admitted to any of the participating hospitals, between March 2020 to March 2022. Individuals who developed first COVID-19 symptoms during hospitalisation or those who were transferred to another hospital not included in the cohort were not considered (Fig. 1).

### Data collection

Data collection was performed from the medical records by healthcare professionals or undergraduate students (from Medical and Nursing schools), properly trained in the research protocol, as detailed elsewhere [15]. The following variables were collected, using the Research Electronic Data Capture (REDCap) tools [17, 18]: (i) demographic data and previous clinical history; (ii) clinical assessment upon hospital presentation; (iii) laboratory findings; and (iv) outcomes (for more details, see Supplementary File 1).

Since our study involved data collected from medical records, diagnosis of previous dementia was based on clinical reports, ongoing treatment for dementia, or information provided by family members or caregivers, which were collected upon patient admission to the hospital. In Brazil, the diagnosis of dementia follows the criteria recommended by the Brazilian Academy of Neurology [19] and the Clinical Protocol and Therapeutic Guidelines for Alzheimer's disease proposed by the Ministry of Health of Brazil [20], based on the criteria for the clinical diagnosis of Alzheimer's disease established by the National Institute of Neurological and Communicative Disorders and Stroke (NINCDS) and the Alzheimer's Disease and Related Disorders Association (ADRDA) [21]. The etiology, biomarkers, and stage of previous dementia were not collected. Delirium at hospital presentation was assessed as a report of delirium, or abnormal mental state (such as confusion, torpor, obnubilation, agitation, rapid mood changes or hallucinations, and refusal to cooperate with care), or Glasgow Coma Score lower than 15.

To ensure data quality, the database was regularly audited. All the outliers and missing information were reviewed and corrected by local references from each hospital.

### Outcomes

The primary outcome was in-hospital mortality. Secondary outcomes included hospital length of stay, admission, and length of stay in the intensive care unit (ICU), invasive mechanical ventilation (IMV) support, kidney replacement therapy (KRT), the incidence of sepsis, nosocomial infection, and thromboembolic events.

### Sample size

Since all patients who fulfilled the inclusion criteria were selected to compose the sample, a prespecified sample size was not calculated.

### Statistical analysis

This study undertook a matched analysis comparing patients with preexisting dementia to controls using propensity score matching. We employed a logistic regression model to estimate the propensity score. This model incorporated predictors such as age, sex, comorbidities (including arterial hypertension, diabetes mellitus, stroke, obesity, heart failure, and cancer), study center, and admission year (2020 vs. 2021–2022), using dementia as the outcome. Since we used the propensity score to match cases and controls, we did not conduct additional multivariate analyses. Patients from the control group were searched to find those who had the closest propensity score from the study group (within 0.17 standard deviations of the logit of the propensity score, on a scale from 0–1.00), using the MatchIt package in R software. For each patient with dementia, the software selected one to three patients without dementia, matched by the variables previously described.

Categorical data were presented as numbers and proportions, while continuous variables were expressed as median and interquartile range (IQR). The Wilcoxon or t-tests were used to compare continuous variables—according to data distribution, and Chi-Square or Fisher Exact tests for categorical variables.

Statistical tests were conducted with an alpha level of 0.05 in two-sided tests, considering a *p*-value ≤ 0.05 as statistically significant. All analyses were performed using R software (version 4.0.2).

## Results

**Fig. 2:**
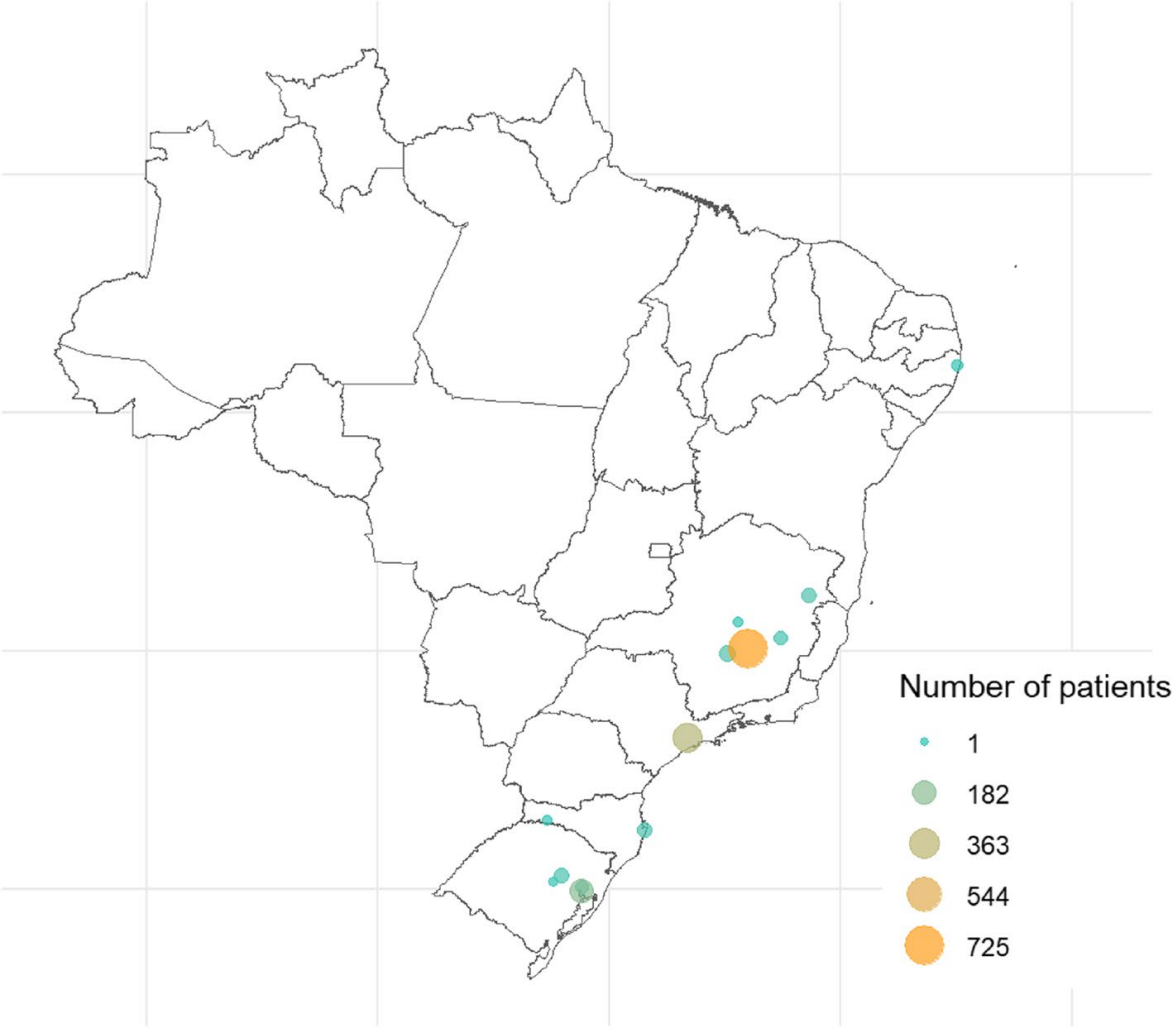
Map of Brazil indicating the city of residence of the study patients. Geobr version 1.7.0; ggrepel version 0.9.3; leaflet version 2.1.2. https://www.r-project.org/

Of 1,556 patients included in the study, 405 had a diagnosis of dementia and 1,151 were matched controls (Fig. 1). The median age was 82.0 (IQR 76.0–87.0) years old, and 58.7% were women. Figure 2 shows the city of residence of patients in this study.

Demographic and clinical characteristics were similar between the groups, except for previous smoking, which was less frequent in the dementia than in the control group (14.3% vs. 21.3%, *p* = 0.003) (Table 1).

**Table 1:** Baseline demographics and clinical characteristics of the COVID-19 patients with dementia and matched controls without dementia

Variables	Dementia *N* = 405^1^	Control patients^a^ *N* = 1,151^1^	*p*-value^2^
Age (years)	82.0 (76.0, 87.0)	81.0 (75.0, 87.0)	0.311
Men	166 (41.0%)	481 (41.8%)	0.823
***Comorbidities***			
Arterial hypertension	242 (59.8%)	737 (64.0%)	0.141
Diabetes mellitus	131 (32.3%)	382 (33.2%)	0.803
Stroke	58 (14.3%)	141 (12.3%)	0.324
Heart failure	49 (12.1%)	148 (12.9%)	0.758
Psychiatric disorders	44 (10.9%)	96 (8.3%)	0.154
CKD	42 (10.4%)	112 (9.7%)	0.784
COPD	38 (9.4%)	147 (12.8%)	0.085
CAD	37 (9.1%)	125 (10.9%)	0.377
Atrial fibrillation	34 (8.4%)	86 (7.5%)	0.624
Cancer	24 (5.9%)	66 (5.7%)	0.985
Obesity	21 (5.2%)	53 (4.6%)	0.737
Asthma	14 (3.5%)	46 (4.0%)	0.738
Rheumatologic disease	4 (1.0%)	24 (2.1%)	0.226
Cirrhosis	3 (0.7%)	9 (0.8%)	> 0.999
***Smoking***			
Current smoking	12 (3.0%)	41 (3.6%)	0.680
Previous smoking	58 (14.3%)	245 (21.3%)	0.003

*CAD* coronary artery disease, *CKD* chronic kidney disease, *COPD* chronic obstructive pulmonary disease

^1^n (%); Median (IQR)

^2^Pearson's Chi-squared test; Wilcoxon rank sum test; Fisher's exact test

^a^Matched controls (age, sex, comorbidities [arterial hypertension, diabetes mellitus, stroke, obesity, heart failure, and cancer], hospital, and year of admission)

Patients with dementia had a lower frequency of dyspnoea (58.8% vs. 65.2%, *p* = 0.025), cough (53.6% vs. 59.4%, *p* = 0.046), myalgia (9.4% vs. 21.0%, *p* < 0.001), headache (3.0% vs. 10.4%, *p* < 0.001), anosmia (1.2% vs. 5.6%, *p* < 0.001), and ageusia (1.0% vs. 5.7%, < 0.001) compared to those without dementia. Upon hospital presentation, they exhibited a higher prevalence of delirium (38.5% vs. 22.5%; *p* < 0.001), fever (49.4% vs. 41.7%; *p* = 0.009), sensory impairment (57.3% vs. 25.6%; *p* < 0.001); and a lower frequency of invasive mechanical ventilation (IMV) support (23% vs. 37.8%; *p* < 0.001) compared to the non-dementia counterparts. They also were more eligible for palliative care than the matched controls (39.8% vs. 19.7%, *p* < 0.001) (Table 2). There were no clinically relevant differences in laboratory results (Table S1).

**Table 2:** Clinical characteristics of COVID-19 patients with dementia and matched controls without dementia upon hospital presentation

Variables	Dementia *N* = 405^1^	Control patients^a^ *N* = 1,151^1^	*p*-value^2^
***Self-reported symptoms***			
Duration of symptoms (days)	5.0 (3.0, 7.0)	7.0 (4.0, 10.0)	< 0.001
Dyspnoea	238 (58.8%)	750 (65.2%)	0.025
Cough	217 (53.6%)	684 (59.4%)	0.046
Fever	200 (49.4%)	480 (41.7%)	0.009
Delirium	156 (38.5%)	259 (22.5%)	< 0.001
Adynamia	132 (32.6%)	364 (31.6%)	0.766
Diarrhoea	41 (10.1%)	156 (13.6%)	0.089
Rhinorrhoea	41 (10.1%)	130 (11.3%)	0.578
Myalgia	38 (9.4%)	242 (21.0%)	< 0.001
Headache	12 (3.0%)	120 (10.4%)	< 0.001
Ageusia	4 (1.0%)	66 (5.7%)	< 0.001
Anosmia	5 (1.2%)	64 (5.6%)	< 0.001
***Clinical assessment***			
Sensory impairment	232 (57.3%)	295 (25.6%)	< 0.001
HR (bpm)	84.0 (73.0, 94.0)	82.0 (73.0, 93.0)	0.740
RR (bpm)	22.0 (19.0, 24.0)	21.0 (19.0, 25.0)	0.914
Mechanical ventilation	93 (23.0%)	435 (37.8%)	< 0.001
Systolic blood pressure (SBP)			0.150
SBP ≥ 90 mmHg	360 (93.0%)	959 (90.4%)	
SBP < 90 mmHg	9 (2.3%)	22 (2.1%)	
Inotropic requirement	18 (4.7%)	80 (7.5%)	
Diastolic blood pressure (DBP)			0.127
DBP > 60 mmHg	295 (76.4%)	798 (75.4%)	
DBP ≤ 60 mmHg	73 (18.9%)	180 (17.0%)	
Inotropic requirement	18 (4.6%)	80 (7.6%)	
SF ratio	342.9 (248.0, 428.6)	335.7 (217.5, 433.3)	0.261
Palliative care	161 (39.8%)	227 (19.7%)	< 0.001

^1^Median (IQR); n (%)

^2^Inotropic use at admission. bpm: Beats per minute; HR: Heart rate; RR: Respiratory rate; SF ratio: Peripheral capillary oxygen saturation/fraction of inspired oxygen (SpO_2_/FiO_2_ Ratio)

^a^Matched controls (age, sex, comorbidities [arterial hypertension, diabetes mellitus, stroke, obesity, heart failure, and cancer], hospital, and year of admission)

During the hospital stay, patients with dementia had a lower frequency of corticoids usage (68.1% vs. 77.3%, *p* < 0.001), vasoactive amine requirement (22.2% vs. 35.6%, *p* < 0.001), and neuromuscular-blocking drug (4.8 vs. 11.2%, *p* = 0.026) than patients without dementia. The usage of other medications was similar between the groups (Table 3).

**Table 3:** Medications used by COVID-19 patients with dementia and matched controls without dementia during hospitalisation

Variables	Dementia *N* = 405^1^	Control patients^a^ *N* = 1,151^1^	*p*-value^2^
Anticoagulants	294 (72.6%)	882 (76.8%)	0.106
Antibiotics during acute phase of COVID-19	294 (72.6%)	872 (75.8%)	0.231
Antibiotics for nosocomial infection	277 (68.4%)	795 (69.2%)	0.814
Corticoids	276 (68.1%)	888 (77.3%)	< 0.001
Vasoactive amines	90 (22.2%)	410 (35.6%)	< 0.001
Oseltamivir	77 (32.1%)	231 (34.7%)	0.507
Nonsteroidal anti-inflammatory drugs	29 (18.8%)	53 (12.5%)	0.075
Inhaled corticoids	24 (14.5%)	78 (16.1%)	0.723
Statin	21 (13.6%)	61 (14.4%)	0.917
Antiarrhythmic	10 (6.5%)	47 (11.1%)	0.137
Antifungal	10 (3.1%)	46 (5.1%)	0.204
Hydroxychloroquine	9 (5.8%)	31 (7.3%)	0.663
Neuromuscular-blocking drug (except for intubation)	8 (4.8%)	54 (11.2%)	0.026
Clarithromycin	7 (4.5%)	22 (5.2%)	0.918
Convalescent plasma	2 (0.6%)	11 (1.2%)	0.533
Chloroquine	0 (0.0%)		0.122
Tocilizumab	0 (0.0%)	7 (0.8%)	0.200
Immunoglobulins	0 (0.0%)	4 (0.3%)	0.578
Interferon	0 (0.0%)	2 (0.5%)	> 0.999
Remdesivir	0 (0.0%)	1 (0.1%)	> 0.999
Favipiravir	0 (0.0%)	0 (0.0%)	
Ritonavir/Lopinavir	0 (0.0%)	0 (0.0%)	
Umifenovir	0 (0.0%)	0 (0.0%)	
Other specific therapy instituted for COVID-19	21 (6.6%)	73 (8.0%)	0.475
No specific therapy instituted for COVID-19	86 (27.0%)	215 (23.7%)	0.269

^1^n (%); Median (IQR)

^2^Pearson's Chi-squared test; Wilcoxon rank sum test; Fisher's exact test

^a^Matched controls (age, sex, comorbidities [arterial hypertension, diabetes mellitus, stroke, obesity, heart failure, and cancer], hospital, and year of admission)

Regarding patients’ outcomes, those with dementia had lower frequency of admission (32.7% vs. 47.1%, *p* < 0.001) and shorter length of stay (7.0 vs. 9.0 days, *p* = 0.026) into the ICU, and lower frequency of sepsis (17.0% vs. 24.0%, *p* = 0.005), KRT (6.4% vs. 13.0%, *p* < 0.001), and IMV support (4.6% vs. 9.8%, *p* = 0.002), when compared to the control group. We did not observe differences in hospital mortality between patients with and without dementia (Table 4).

**Table 4:** Comparison of outcomes during hospital stay among patients with dementia and matched controls without dementia

Variables	Dementia *N* = 405^*1*^	Control patients^a^ *N* = 1,151^1^	*p*-value^2^
Hospital length of stay	9.0 (5.0, 16.0)	10.0 (6.0, 17.0)	0.115
ICU	132 (32.7%)	542 (47.1%)	< 0.001
Days at ICU	7.0 (4.0, 10.0)	9.0 (4.0, 15.0)	0.026
In hospital mortality	185 (45.7%)	503 (43.7%)	0.528
Acute severe respiratory syndrome	120 (29.6%)	385 (33.4%)	0.177
Nosocomial infection	73 (18.0%)	250 (21.7%)	0.132
Sepsis	69 (17.0%)	276 (24.0%)	0.005
Kidney replacement therapy	26 (6.4%)	150 (13.0%)	< 0.001
Invasive mechanical ventilation	18 (4.6%)	106 (9.8%)	0.002
Pulmonary thromboembolism	15 (3.7%)	51 (4.4%)	0.630
Myocardial infarction	6 (1.5%)	21 (1.8%)	0.815
Venous thrombosis	4 (1.0%)	27 (2.3%)	0.140

^1^Median (IQR); n (%)

^2^Wilcoxon rank sum test; Pearson's Chi-squared test; Fisher's exact test. ICU: Intensive care unit

^a^Matched controls (age, sex, comorbidities [arterial hypertension, diabetes mellitus, stroke, obesity, heart failure, and cancer], hospital, and year of admission)

## Discussion

In this large Brazilian cohort, we found that older adults with dementia hospitalised for COVID-19 showed different clinical presentations and outcomes compared to a matched sample of patients without dementia. Patients with dementia had lower duration and lower frequency of COVID-19 symptoms, including dyspnoea, cough, myalgia, headache, ageusia, and anosmia. However, they had a higher prevalence of delirium upon hospital presentation than the control group. Furthermore, patients with dementia were more frequently referred for palliative care and were less likely to be admitted to the ICU and use invasive support therapy (e.g., IMV support, KRT). Interestingly, we did not observe differences in hospital mortality comparing patients with and without dementia, showing that this diagnosis alone should not be used to predict COVID-19 prognosis.

Patients with dementia are frequently unable to describe their symptoms, given the impaired awareness and communication, which probably contribute to postponing the diagnosis and may explain our findings [22]. Additionally, some studies have demonstrated a high frequency of atypical presentations in older adults with COVID-19, especially in those with dementia [23]. In our study, delirium at hospital presentation had a higher incidence in those with dementia than those without this syndrome. In fact, delirium has been described as an atypical symptom of COVID-19, particularly in frail older adults and patients with dementia [24]. Delirium and dementia frequently coexist, and preexisting dementia is considered an important risk factor for delirium [25]. Clinicians should be aware of such atypical presentations of COVID-19 in older patients with dementia, leading to a lower threshold for testing and introduction of isolation measurements.

Surprisingly, our results showed that patients with dementia had similar hospital mortality compared to matched controls without dementia. On the contrary, two systematic reviews and meta-analyses showed that individuals with dementia presented a higher risk of death than those without dementia [5, 26]. It is worth mentioning that these previous studies included only patients infected in 2020, which means they limited their findings to periods prior to the availability of vaccines. Furthermore, patients from South America were not considered, despite this region being severely hit by the COVID-19 pandemic and having one of the highest rates of dementia globally. Another problem was the high heterogeneity among the studies included in these reviews. Moreover, there was no adjustment for age and comorbidities, which are important confounders in studies involving patients with dementia. For instance, a cohort study comprising patients of the UK Biobank did not show dementia as a risk factor for mortality after COVID-19 in patients younger than 80 years old [8].

Therefore, basing the therapeutic and prognosis of COVID-19 solely on the previous diagnosis of dementia seems inaccurate and premature, as dementia should be considered in light of other risk factors. For example, frailty has been proposed as a key element of risk in the context of COVID-19. In line with this concept, the CO-FRAIL Study [13], conducted in one of the centers included in the present analysis, determined that frailty, assessed using the Clinical Frailty Scale (CFS) [27], was associated with mortality in patients hospitalised due to COVID-19. The authors demonstrated that frailty was able to identify different mortality risks within patients of similar age and similar levels of acute morbidity [13]. Dementia and frailty are closely related conditions in older adults, often sharing common etiological pathways. Furthermore, a high prevalence of frailty syndrome is often observed in older people with dementia. However, identifying frailty in patients with dementia involves considering additional factors, such as multimorbidity, sensory deficits, physical impairment, fatigue, weight loss, and a history of falls [28]. A comprehensive frailty assessment, capturing risks beyond those associated with specific comorbidities like dementia, can offer valuable prognostic information for COVID-19 patients [13, 29]. Therefore, incorporating a frailty measure into the medical assessments of older COVID-19 adults with dementia might aid clinicians in making informed decision-making for these patients.

During the hospital stay, having a diagnosis of dementia was associated with a lower admission and the length of stay in the ICU, sepsis, KRT, and IMV support. Consistent with these results, patients with dementia received fewer vasoactive amines, corticoids, and neuromuscular-blocking drugs than patients in the control group. These medicines are frequently required by patients in intensive care treatment, especially those in mechanical ventilation. Nevertheless, on admission, the dementia group had less frequency of mechanical ventilation support than the control group, which could reflect a less severe acute disease in patients with dementia. Given that the mortality rate was similar between both groups and the dementia group was more likely to receive palliative care support, it is plausible that promoting well-being, aligning with the principles of palliative care, prioritising patient comfort, and dignity [13]. A recent published meta-analysis did not demonstrate an association between frailty status and short-term mortality in patients hospitalised with COVID-19, after adjusting for patient age. This meta-analysis showed that frail patients, compared to non-frail ones, were commonly less admitted to ICU and had less IMV support, which suggests that frailty was a significant factor considered when indicating intensive care therapy [30]. However, in another meta-analysis, Subramaniam et al. showed that frail patients with COVID-19 were commonly admitted to ICU, had greater hospital mortality, and spent fewer days in ICU. Frail patients requiring IMV had a greater risk of death than non-frail patients [31]. Nevertheless, even though frailty and dementia are strongly related, other risk factors for frailty, beyond dementia, should be assessed to evaluate frailty in individuals with dementia. In conjunction with our findings, these results suggest that the decision-making for older adults with COVID-19 should factor in other considerations, including frailty and disease severity, beyond the mere presence or absence of dementia.

### Limitations and strengths

This study has limitations. This is a retrospective study, which inherently carries limitations of medical records review. The possibility of underdiagnosis of dementia in the hospital setting cannot be excluded, potentially leading to information bias concerning our primary predictor. Additionally, the specific etiology, biomarkers, and stage of dementia were not specified in this study. While we included many Institutions from Brazil, the participating hospitals may not fully represent all regions in the country. As previously mentioned, we did not analyze frailty in older adults hospitalised with COVID-19. Moreover, patients in the dementia group had a lower frequency of mechanical ventilation support than the control group on admission to the hospital, which could reflect a less severe acute disease in these patients.

On the other hand, this study has important strengths. It is a multicentre cohort involving several hospitals, from different cities in Brazil. Additionally, it encompasses the pandemic waves with various virulent strains of the virus and covers both pre- and post-vaccination for SARS-CoV-2 in Brazil [32]. We employed the propensity score matching to balance potential confounders (age, sex, comorbidities, periods of pandemic, and the hospitals included in the cohort). The groups were similar considering all demographic and clinical characteristics, except for previous smoking, which was less prevalent in the dementia group. However, we acknowledge that this variable could be influenced by memory bias in the dementia group. Finally, we conducted a comprehensive analysis of patients’ characteristics and the in-hospital outcomes.

Finally, understanding optimal approaches to the prevention and management of COVID-19 is crucial for preventing future infections and deaths among inpatients during the resurgence of this or any other pandemic [6]. Our results raise important questions that could influence the management of older COVID-19 adults with dementia.

## Conclusion

In comparison to matched controls, patients with dementia had a lower frequency of dyspnoea, cough, myalgia, headache, ageusia, and anosmia; but a higher frequency of fever and delirium. Additionally, they had a lower frequency of ICU admission, sepsis, KRT, and IMV support. However, there was no difference in in-hospital mortality. Importantly, our data indicates that dementia, by itself, does not correlate with an increase in hospital mortality among COVID-19 patients. Overall, our results suggest the importance of understanding the unique manifestations of COVID-19 in patients with dementia. This understanding can guide and enhance the medical management of these patients, including decisions related to intensive care. Further studies are needed to explore and refine the role of other prognostic risk factors in older adults with dementia hospitalised with COVID-19.

### Supplementary Information

**Additional file 1.** Guidance manual for data collection.

**Additional file 2: Table S1. **Laboratory exams among the included COVID-19 patients with dementia and matched controls without dementia.

## Data Availability

The datasets used and/or analysed during the current study are available from the corresponding author on reasonable request.

## Abbreviations

ADRDA: Alzheimer’s Disease and Related Disorders Association
AST: Aspartate transaminase
ALT: Alanine aminotransferase
CAAE: Certificado de Apresentação de Apreciação Ética
CAD: Coronary artery disease
CFS: Clinical Frailty Scale
CKD: Chronic kidney disease
CO-FRAIL: COVID-19 and Frailty Study
COPD: Chronic obstructive pulmonary disease
COVID-19: Coronavirus disease 2019
DBP: Diastolic blood pressure
HR: Heart rate
ICU: Intensive care unit
IMV: Invasive mechanical ventilation
INR: International normalised ratio
IQR: Interquartile range
KRT: Kidney replacement therapy
NINCDS: National Institute of Neurological and Communicative Disorders and Stroke
PaO_2_: Partial pressure of oxygen
PaCO_2_: Partial pressure of carbon dioxide
REDCap: Research Electronic Data Capture
RR: Respiratory rate
SARS-CoV-2: Severe acute respiratory syndrome coronavirus 2
SF ratio: Peripheral capillary oxygen saturation/fraction of inspired oxygen (SpO_2_/FiO_2_ Ratio)
STROBE: Strengthening the Reporting of Observational Studies in Epidemiology
SBP: Systolic blood pressure
TGO/AST: Aspartate aminotransferase
TGP/ALT: Alanine aminotransferase
