# Supplementary material for: Clinical characteristics and outcomes of COVID-19 patients with preexisting dementia: a large multicenter propensity-matched Brazilian cohort study
Source: BMC Geriatr. 2024 Jan 5;24:25. doi: 10.1186/s12877-023-04494-w (PMC10770897; doi:10.1186/s12877-023-04494-w)
Supplement: Supplementary file 1 — Additional file 1. Guidance manual for data collection. [file 12877_2023_4494_MOESM1_ESM.pdf]

Supplementary File 1.

Federal University of Minas Gerais

Brazilian Multicentre Hospital Registry of Patients with COVID 19

GUIDANCE MANUAL FOR DATA COLLECTION

Project Coordinated by:

Dr Milena Soriano Marcolino, Federal University of Minas Gerais

Manual Prepared by:

Israel Borges, Karina Prado, Karen Ruschel, Luanna Monteiro, Maíra Viana, Milena Soriano Marcolino, Talita Fischer, Thais Sales

Belo Horizonte, 2020

List of Abbreviations

CVA	Cerebral Vascular Accident (Stroke)
BAV	Atrioventricular Block
BAVT	Total Atrioventricular Block
bpm	Beats per Minute
ARB	Angiotensin receptor blocker
NC	Nasal Catheter
COVID-19	Coronavirus Disease 2019
MRS	Myocardial Revascularisation Surgery
CTI	Intensive Care Unit
D3	Day 3
D5	Day 5
CPOD	Chronic Obstructive Pulmonary Disease
ACE	Angiotensin Converting Enzyme
ECG	Electrocardiogram
ECMO	Extracorporeal Membrane Oxygenation
LVEF	Left Ventricular Ejection Fraction
FiO_2	Fraction of Inspired Oxygen
LMWH	Low Molecular Weight Heparin
HCO_3^-	Bicarbonate
HIV	Human Immunodeficiency Virus
UFH	Unfractionated Heparin
IgM	Immunoglobulin M
BMI	Body Mass Index
Inc/min	Respiratory Incursions per Minute
LABA	Long-Acting Beta2 Agonist
LAMA	Long-Acting Muscarinic Antagonist
LDH	Lactate Dehydrogenase
FM	Face Mask
NR	Not Performed
O_2	Oxygen
pCO_2	Partial Carbon Dioxide Pressure
CRP	C-Reactive Protein
pH	Potential of Hydrogen
IBP	Intra-Arterial Pressure (invasive arterial pressure)
pO_2	Partial Oxygen Pressure
aPPT	Activated Partial Prothrombin Time
QTc	Corrected QT Interval

RNI	International Normalised Ratio
RT-PCR	Reverse transcription polymerase chain reaction
RX	X-Ray
SAPS-3	Simplified Acute Physiological Score
ARDS	Acute Respiratory Distress Syndrome
SARS-COV-2	Severe Acute Respiratory Syndrome - coronavirus 2
SC	Subcutaneous
SDRA	Acute Respiratory Distress Syndrome
SOFA	Sequential Organ Failure Assessment
SV	Supraventricular
TC	Tomography
TEP	Pulmonary Thromboembolism
AST/GOT	Aspartate Aminotransferase / Glutamic Oxalacetic Transaminase
ALT/GPT	Alanine Aminotransferase / Glutamic Pyruvic Transaminase
V-tach	Ventricular Tachycardia
DVT	Deep Vein Thrombosis
LV	Left Ventricle
IMV	Mechanical Ventilation

Presentation

This manual for completion of the **form** on the electronic platform REDCap® was created to help the applicant, to make this filling-in process easier and quicker. This is a practical tool, in which the main questions on the form are explained in detail, so that the applicant may easily find the instructions and immediately clarify any doubts with regard to the possibilities of responses and the correct way to fill in the form.

Instructions for filling in the questions follow the structure of the **form** and the order of the questions. With this manual in hand, it is possible to find out in advance about the main questions and also consult it if any doubts arise during the application.

The completion of the form, following this manual, is essential to allow the identification of outcomes and to ensure the quality of the outcomes and ensure the quality of the evidence originated from the study.

Objectives

The goal of this **guidance manual** is to present the content of the form of the REDCap® electronic platform for the standardisation of the data collection process.

The form, which is the source object of this manual, is the instrument used for the "**Brazilian Multicentre Hospital Registratry of Patients with COVID-19"** as approved by the ethics committee CONEP (CAAE 30350820.5.0000.0008).

Targeted Audience

At this phase, will be eligible patients admitted to hospital institutions (through self-request, service transfer or referred by prehospital service), with a confirmed COVID-19 diagnosis by detectable RT-PCR or rapid test (antigen or IgM serology), from March 30th to December 31st 2021.

Data Collection

Records from eligible patients will be selected and data regarding hospital admission, hospitalization period and hospital discharge or death. Also, in the new phase of the project post-hospital discharge data will be collected at the time of discharge, after two months, after four and six months. **The present manual exclusively regards hospital records. Post-hospitalization data collection will be focused on a specific document.**

Availability and Period of Application

Data will be collected through an electronic platform (REDCap®). Data collection researchers will access the platform through the link http://telessaude.hc.ufmg.br/cursos/AcessoRedCap.php.

After registration, the project's coordination will enable access via email. Researchers from each institution will have access to patients from their designated institutions only. Each researcher must use their own password.

Data from eligible patients will be included in the database after a local ethic's committee or the institution's approval, until December 31st 2021.

Place of Application

Forms will be filled in the participation institutions.

Responsibility for Filling In the Form

The **form** will be filled out by researchers from each institution assigned to the project, duly trained in the study protocol and application manual. Data will be monitored by the registry coordination and, in case of questions, local researchers will be contacted.

Application time

The time it takes to fill out the **form** is variable. The average time is estimated to be 40 minutes for each patient.

Questions

The form is composed of 205 variables, divided into four collection moments (hospital admission, hospitalization, and hospital discharge/death).

Methods

<u>**Preparation**</u>

Applicators must access the **form** on the REDCap® electronic platform.

The **form** will be completed using data from the **medical records** of patients admitted to partner hospitals (by reading and extracting data from medical records), the **exams system** (in many institutions, not all exams are copied into medical records) and **prescriptions**, **retrospectively**.

Before starting data collection, make sure you are able to put into practice all the guidelines provided during the training conducted by the project's technical team. If you have any questions, please contact the project staff.

The following are general guidelines on how to proceed during the evaluation of medical records. These guidelines are essential to guide the researcher's conduct during data collection:

- Do not make any changes to the records (do not enter new information and do not delete existing information).
- In cases of data collection in physical medical records, contact the person responsible for the medical record collection/system in the institution and schedule times for data collection in advance.
- Remember to have the institution's letter of consent at hand in case clarification is required regarding your access to the medical records. Make yourself available to answer any questions related to the execution of the project at the institution.
- For data collection from the electronic medical record, make sure your access is active in the institution.

Data Collection Guidelines

Form Number

It is an automatic number, assigned by REDCap®. If a researcher from a given center fills out the REDCap® off-line and another researcher fills out online, the same form numbers can be assigned, in principle, and when importing the data off-line to online, the form numbering can be changed.

Center Numbering

Each research center will have a corresponding 4-digit number (center no.). Make sure you have your center number before you start collecting data. If you have any questions, please contact the project's technical team.

Identification number (ID) in the study

The patient number (study ID) is a seven digit field consisting of the center number (4 digits) appended by a sequential patient identifier (3 digits): Example: 1001001 is the first subject number of center 1001. **In this case, we note again the importance of sharing a drive spreadsheet among all the center's researchers responsible for data collection, so that there is no duplication of identification numbers assigned to patients during their inclusion in REDCap®.**

Filling out the form

The form is divided into three collection moments:

1. Hospital Admission: includes primary identification and past history, clinical evaluation on admission, and laboratory tests performed within 24 hours of admission;

2. Hospitalization: includes clinical evaluation and laboratory tests upon ICU admission, instituted therapy, and supportive care throughout the hospitalization period;
3. Hospital discharge or death: and outcomes.

Type of answer option
1. Required: this variable must be filled out for all patients in all situations.
2. Not mandatory: whenever possible, avoid leaving unanswered.
3. Personalized information: variable with a free text field, to be filled out according to the details described in the guidelines for filling out. It can be mandatory or non-mandatory.
4. Conditioned: variable will be available according to the answer to the previous question. Example: when selecting "Sex: female", the variable "Pregnant" will be accessible, to specify the woman's condition. It can be **required or not mandatory.**

It is important to fill out the form with as much information as possible. At the end of each step of the form, there is a question about "form status - complete?", with options "incomplete", "unverified" and "complete". After filling in all the available data, at the end of the form, update the status to "complete". If it is not possible to complete the form completely at that time, select "incomplete". If you need to review the completed form at another time, you can mark it as "unverified". This choice will determine the color assigned to each step of the form on the "record home page", green, red, and yellow, respectively.

The variables are detailed below:

Moment 1: Hospital Admission

Form 1: Primary identification and past history

**Primary Identification**			
**Nº**	**Item**	**Answer Option**	**Guidelines for filling out**
1	Study ID	Required	Fill in the **four digits referring to the center number**, followed by **three digits**, which represent the **patient's identifier** in the institution, which must be **sequential**
2	Name Initials	Required	Fill in the **initials of the first name and the initials of the second and last and last surnames** (ex. José Antonio da Silva, use JAS, for first and last name)
3	Method of COVID-19 confirmation	Required	Fill in: 1- RT-PCR; 2- Rapid antigen test; 3- Rapid serological test; 4- Rapid test not specified. This question accepts more

			than one answer.<br>This study only includes patients with confirmed Covid-19.<br>If the patient does not have confirmed Covid-19, end questionnaire.
4	Medical record	Required	Fill in the patient's medical record number
5	Birth date	Required	Enter in the format DD/MM/YYYY or select the date in the calendar.
6	Sex at birth	Required	Select one of the options: female or male
6.1	Pregnancy	Conditioned/ required	Select the yes or no option. If you select the option option in the item sex at birth, this variable will be inactive.
6.1.1	Weeks of pregnancy?	Conditioned/ personalized information	Enter the number of weeks. If you selected no in the previous item, or if selected the male option in the item sex at birth, this variable will be inactive
7	Date of admission	Required	Fill in the date of admission to the current institution. **If the patient was admitted for another reason and during the course<br>the course of the hospitalization he/she starts to develop symptoms of symptoms, enter the actual admission date (it does not have to be the<br>be the date of onset of Covid-19 symptoms). This has been<br>changed from the first phase of the project.**
7.1	Transferred from another service?	Required	Select the option related to the patient's situation, among: 1 - No; 2 - Emergency care unit; 3 - Hospital in the same city; 4 - Hospital in another city; 5 - Long-stay facility; 6 - Field hospital; 7- No information
8	City of residency	Required	Fill out with the patient's municipality of origin

**Past History***			
**Nº**	**Item**	**Answer Option**	**Guidelines for filling out**
9	Received COVID-19 vaccine?	Required	Select the option yes or no. If you select the option that<br>received vaccine, it will open 3 questions, as follows:

9.1	Which vaccine?	Required	Select the option referring to the vaccine(s) taken by the patient patient: 1-Astrazeneca; 2-Coronavac; 3-Janssen; 4- Pfizer; 5- Sputnik; or 6-No information
9.1.1	Which other vaccine?	Conditioned	Describe the name of the other vaccine if it is not included in the previous item.
9.2	How many doses?	Required	Select the appropriate option: 1-One; 2-Two; 3-Three; 4-No information
9.3	Date of the last dose	Conditioned	Fill in the date of the last dose of the Covid-19 vaccine. **If available the month, but not the specific day**, consider the 15th (as an approximation, since it is the middle of the month).
10	Cardiovascular system	Required	Select the option(s) for the patient (multiple answers accepted) multiple answers): 1-Hypertension; 2-Coronary artery disease; 3- Heart failure; 4-Atrial fibrillation/flutter; 5-Ischemic stroke ischemic stroke; 7- Previous venous thromboembolism Chagas' disease; 7-Other cardiovascular diseases. other cardiovascular diseases; 8-None relevant disease
10.1	Describe other cardiovascular disease	Conditioned	When selecting the option "other cardiovascular disease" in the previous question, specify here the disease (free text). If the option "other cardiovascular disease" option is not selected, this item will not be available.
11	Respiratory system	Required	Select the options referring to the patient (multiple answers accepted): 1- Asthma; 2- COPD; 3-Pulmonary fibrosis; 4- Active tuberculosis (under treatment or untreated); 5- Tuberculosis treated in the past (already completed the treatment); 6-None of the above.
12	Metabolic disease	Required	Select the option(s) related to the patient (multiple answers accepted) multiple answers): 1 - Diabetes Mellitus; 2 - Obesity (BMI > 30kg/m2); 3 - None of the above.
13	Other health conditions	Required	Select the option(s) related to the patient (multiple answers accepted) multiple answers): 1 - Cirrhosis; 2-Dementia; 3 - Psychiatric disease; 4 - Chronic renal disease; 5 -

			Rheumatologic/connective tissue disease; 6 - Thyroid disease; 7 - HIV infection - HIV infection; 8 - Malignant neoplasm; 9 - Postpartum <6 weeks; 10 - Prior transplantation; 11 - Other relevant condition; 12 - No other relevant health condition.
13.1	Need for dialysis prior to COVID-19?	Conditioned	In case you selected the option "Chronic renal disease" in "Other health conditions", inform if the patient was on dialysis dialysis previously to the illness by COVID-19. Fill in with the option yes or no.
13.2	Which thyroid disease?	Conditioned	In case you have selected the option "Thyroid disease" in the item "Other health conditions", inform which disease: 1- Hypothyroidism; 2- Hyperthyroidism; 3- Other disease; 4- No other disease
13.3	HIV on treatment?	Conditioned	If you have selected the option "HIV infection" in the item "Other health conditions", inform if the patient is under treatment treatment with antiretroviral drugs. Select the option: 1-Yes; 2- No; 3-No information.
13.3.1	Viral Load available? If yes please describe	Conditioned	If you have selected the option "HIV Infection" in the item "Other health conditions", describe the patient's last available viral load available from the patient if it is recorded in the medical record
13.3.2	CD4 Count count available? If yes, please describe	Conditioned	If you have selected the option "HIV infection" in the item "Other health conditions" option, describe the patient's last available CD4 count if it is recorded in the patient's medical record
13.4	Type of cancer	Conditioned	If you have selected the option "Malignant neoplasm" in the item "Other health conditions", select the option referring to the type of neoplasm: 1 - Hematological; 2 - Solid organs with metastases metastasis; 3 - Solid organs without metastasis (or without information about metastasis); 4- Without information about the type of cancer
13.4.1	Primary site of the cancer	Conditioned	In case you have selected the option "Malignant neoplasm" in the item

			"Other health conditions", inform primary site of the<br>cancer. If not available, write ND.
13.4.2	Treatment in use<br>for the cancer	Conditioned	In case you have selected the option "Malignant cancer" in the item<br>"Outras condições de saúde" (Other health conditions), inform treatment in use for the neoplasm, as for example radiotherapy<br>chemotherapy (write down the name of the medicine), etc.
13.5	Type of transplant	Conditioned	If you have selected the option "Previous transplantation" in the item<br>"Other health conditions", select the option referring to the<br>type of transplant among: 1 - Hematological; 2 - Solid organ; 3 - No information.
13.5.1	Which organ was transplanted?	Personalized information	In case you have selected the option "Solid organ" in the<br>previous item, inform which organ was transplanted.
13.6	Describe the other<br>health condition	Personalized information	If you selected the option "Other - which? "Other health conditions", fill in the name of the condition<br>in case of a relevant disease.
14	Continuous medications[a]	Required	Select the option(s) for the patient (multiple answers accepted)<br>multiple answers): 1 - Oral anticoagulant; 2 - Inhaled<br>3 - Oral corticoid; 4 - Immunosuppressant; 5 - Does not use any of these<br>uses any of these medications
15	Life habits	Required	Select the option(s) for the patient (multiple answers accepted)<br>multiple answers): 1 - Illicit drugs; 2 - Alcoholism; 3 - Current<br>Current smoker; 4 - Former smoker; 5 - None of the above
16	Functional status	Required	Select the option for the patient **before getting sick** from<br>Covid-19:<br>1- **Robust** - encompasses very active (exercised regularly), active (no active symptoms of illness, exercised<br>exercised occasionally) and regular (health problems well controlled);<br>2- **Vulnerable or mild frail -** not dependent, but slower, tired throughout the day; or needs help with instrumental

			instrumental ADLs (finances, transportation, housework home work, medications);<br>3- **Moderately frail** (needs help with bathing and dressing) help with bathing and dressing);<br>4- **Severely or very severely fragile** (totally dependent dependent on DLAs);<br>5- **Terminally ill** (life expectancy < 6 months);<br>6- No information

*For **past history** purposes, consider the following definitions:

Comorbidities

• **Cardiovascular system:**

o **Hypertension:** previous diagnosis of hypertension reported in medical records and/or the use of hypertensive medication, whether regularly or irregularly treated, controlled or not.

o **Coronary artery disease:** history of previous angioplasty, coronary artery bypass grafting surgery (CABG), acute myocardial infarction, or angina.

o **Heart Failure:** registro em prontuário de insuficiência cardíaca, independente se fração de ejeção preservada ou reduzida.

o **Atrial fibrillation or atrial flutter:** record in the medical chart any atrial fibrillation (paroxysmal or permanent) or atrial flutter.

o **Ischemic stroke:** past history of cerebral ischemia recorded in medical records.

• **Sistema respiratório:**

o **Asthma:** report of asthma or "bronchitis", with previous compatible symptoms in adulthood, regardless of the use of control medication (example: corticoid/LABA), in the case of adults. For pediatric patients, consider asthma if there has already been an asthma attack or "bronchitis" at any time during childhood.

o **COPD:** report of COPD independent of the use of inhaled control medications, but with compatible risk factors (Example: smoking > 20 years/pack, prolonged use of wood stove) OR patients using inhaled medications compatible with COPD treatment (LABA, LAMA, or combination thereof with or without inhaled corticosteroids) and associated risk factor.

o **Pulmonary Fibrosis:** in the medical record.

o **Active tuberculosis:** in the medical record.

o **Treated past tuberculosis:** in the medical record.

• **Metabolic diseases:**

o **Diabetes mellitus:** report in the medical record of diabetes, of any type, insulin applicant or non-insulin applicant.

o **Obesity:** BMI > 30Kg/m²

• **Continuous use medication:** mark the medications that the patient takes according to the medical chart.

• **Life habits** – according to medical records:

o **Alcoholism:** report of drinking in the medical record, unless "social drinking" or up to two standard drinks per day is reported: 2 cans of beer, two shots of cachaça, two glasses of wine, two glasses of whiskey.
o **Past or current smoker:** report in the medical record, regardless of time and quantity.
o **Use of illicit drugs:** report in the medical record, regardless of quantity.

[a]Regarding drugs, the table below lists the main examples of each therapeutic class:

Class	Medication
Oral anticoagulant	Apixaban, Dabigatran, Edoxaban, Rivaroxaban, warfarin.
Oral corticoid	Betamethasone, Dexamethasone, Prednisolone, Prednisone, Deflazacort.
Inhalatory corticoid	Beclomethasone, Budesonide, Ciclesonide, Dexamethasone, Fluticasone, Mometasone, Triamcinolone.
Immunossupressor	Azathioprine, Cyclophosphamide, Cyclosporine, Everolimos, Methotrexate, Mycophenolate Sodium, Mycophenolate Mofetil, Sirolimus, Tacrolimus.

Form 2: Clinical assessment at admission

**Clinical Assessment on Admission**			
**N°**	**Item**	**Options for Answer**	**Guidance for Completion**
17	Date of first symptom onset	Mandatory	Inform the date of onset of first symptom using the DD/MM/YYYY format, or fill in the date using the calendar.
18	Clinical signs and symptoms	Mandatory	Please select the options that apply to the patient (multiple responses are accepted): 1 – Adinamia; 2 – Ageusia (loss of taste); 3 – Anosmia (loss of smell); 4 – Arthralgia; 5 – Headache; 6 – Coryza; 7 – Diarrhoea; 8 – Dyspnoea; 9 – Sore throat; 10 – Fever; 11 – Haemoptysis; 12 – Hyporexia; 13 – Irritability; 14 – Neurological symptoms; 15 – Myalgia; 16 – Nausea and vomiting; 17 – Skin Rash; 18 – Productive Cough; 19 – Dry Cough – 20 -0 No Symptoms; 21 – Others **If there is any record of cough without specification of type (dry or productive), then please mark “others” and then specify “cough” in item 18.2**

18.1	What neurological symptoms?	Conditional	If you have marked “neurological symptoms” in item 18, please describe which ones.
18.2	Which other signs or symptoms?	Conditional	If you have marked “others” in item 18, please describe which ones.
19	Glasgow Coma Scale	Mandatory / Personalised Information	Please fill in with the patient’s Glasgow score at the moment of admission. If not available, then please use ND. **If there is a record on the file of an alert and lucid patient, or use of the abbreviations LOTE, BOTE or LOCV, then consider a Glasgow score of 15.** **If the patient is under continuous sedation, then fill in with NA (not applicable).**
19.1	State / Level of Consciousness	Conditional	If the information about the Glasgow Coma Scale is not complete or not available, then please select the option(s) that best apply to the patient, choosing between: 1 – Lucid and alert; 2 – Confused; 3 - Disoriented; 4 – Drowsy; 5 – In a state of torpor; 6 – Comatose. **If the patient is in a state of continuous sedation, then please mark “comatose”.**
20	Systolic Blood Pressure	Mandatory / Personalised Information	Inform the systolic blood pressure in mmHg. Complete with NA if not available.
20.1	Diastolic Blood Pressure	Personalised Information	Inform the diastolic blood pressure in mmHg. Complete with NA if not available.
21	If there is monitoring of IBP; mean arterial pressure (mmHg)	Personalised Information	If this is a patient with monitoring of invasive arterial pressure, then please inform the mean arterial blood pressure. Put NA if the information is not available.
22	Is there any use of vasoactive amines?	Mandatory	Fill in with either ‘yes’ or ‘no’ at the moment of confirmation of the pressure as registered in the previous items.
23	Heartbeat Frequency	Mandatory/ Personalised Information	Supply the value of the heartbeat frequency in beats per minute (bpm). Put NA if the information is not available.
24	Breathing Frequency	Mandatory/ Personalised Information	Supply the breathing frequency in Inc/min. Put NA if the information is not available.
25	Temperature	Personalised Information	Inform the temperature in ºC. Put NA if the information is not available.
26	Saturation of O_2	Mandatory/ Personalised Information	Inform the level of saturation of O_2 in %. Put NA if the information is not available.
26.1	Environmental Air	Mandatory	Select one of the options, ‘yes’ or ‘no’, according to the supply of O_2 when the saturation of the previous item is registered.
26.2	Nasal Catheter (NC)	Mandatory/ Conditional	<u>If the ‘no’ option is selected in the ‘Environmental Air’ item, then the option of oxygen supply through a NC shall become available.</u> Select one of the options, ‘yes’ or ‘no’
26.3	Flow	Personalised Information	Inform the flow (L/min) of O_2 offered by the nasal catheter. This item shall only be available for completion if ‘yes’ is selected in the previous item. Put NA if the information is not available.
26.4	Face Mask	Mandatory/ Conditional	<u>If the ‘no’ option is selected in the ‘Environmental Air’ and ‘Nasal Catheter’ items, then the option of</u>

			supply of oxygen through FM shall become available. Select one of the options: ‘yes’ or ‘no’.
26.5	Flow	Personalised Information	Inform the flow (L/min) of O_2 offered by the face mask. This item shall only be available if ‘yes’ is selected in the previous item. Put NA if the information is not available.
26.6	Invasive mechanical ventilation	Mandatory/ Conditional	If the ‘no’ option is selected in the ‘Environmental Air’, ‘Nasal Catheter’, and ‘Face Mask’ items, then the option of supply of oxygen through IMV shall become available. Select one of the options: ‘yes’ or ‘no’.
27	Are there records of the SOFA score?	Mandatory	Select one of the options: ‘yes’ or ‘no’.
27.1	What is the value of the SOFA score?	Mandatory/ Personalised Information	If the ‘yes’ option is chosen in the previous item, then this item shall become available. Indicate the value of the SOFA score.
28	Are there records of the SAPS-3 score?	Mandatory	Select one of the options: ‘yes’ or ‘no’.
28.1	What is the value of the SAPS-3 score?	Mandatory/ Personalised Information	Please inform the SAPS-3 score, should there be no SOFA value. This item shall be available if ‘no’ has been informed for the item ‘are there records of the SOFA score?’

**Laboratory Findings on Admission**			
**Nº**	**Item**	**Options for Answer**	**Guidance for Completion**
29	Haemoglobin (g/dL)	Personalised Information	Inform the value of haemoglobin content on the CBC made at the admission of the patient to hospital. Put NA if this laboratory test is not available.
30	Leukocytes (cells/mm^3)	Mandatory/ Personalised Information	Inform the absolute global leukocyte count on the CBC made at the admission of the patient to hospital. Put NA if this laboratory test is not available.
31	Neutrophils (cells/mm^3)	Personalised Information	Inform the value of haemoglobin content on the CBC made at the admission of the patient to hospital. Put NA if this laboratory test is not available.
32	Rods (cells/mm^3)	Personalised Information	Inform the absolute rod count as on the CBC made at the admission of the patient to hospital. Put NA if this laboratory test is not available.
33	Lymphocytes (cells/mm^3)	Mandatory/ Personalised Information	Inform the absolute lymphocyte count as on the CBC made at the admission of the patient to hospital. Put NA if this laboratory test is not available.
34	Platelets (cells/mm^3)	Mandatory/ Personalised Information	Inform the absolute platelet count as in the test made at the admission of the patient to hospital. Put NA if this laboratory test is not available.
35	Albumin (g/dL)	Personalised Information	Inform the albumin value as in the test made at the admission of the patient to hospital. Put NA if this laboratory test is not available.
36	Total Bilirubin (mg/dL)	Mandatory/ Personalised Information	Inform the total bilirubin value as in the test made at the admission of the patient to hospital. Put NA if this laboratory test is not available.

37	Direct Bilirubin (mg/dL)	Personalised Information	Inform the direct bilirubin value as in the test made at the admission of the patient to hospital. Put NA if this laboratory test is not available.
38	BNP (pg/ml)	Personalised Information	Inform the BNP value as in the test made at the admission of the patient to hospital. Put NA if this laboratory test is not available.
39	Ionic Calcium (mg/dL)	Personalised Information	Inform the Ionic Calcium value as in the test made at the admission of the patient to hospital. Put NA if this laboratory test is not available.
40	Creatinine (mg/dL)	Mandatory/ Personalised Information	Inform the creatinine level as in the test made at the admission of the patient to hospital. Put NA if this laboratory test is not available.
41	Creatinophosphokinase (CPK – U/L)	Mandatory/ Personalised Information	Inform the CPK Value as in the test made at the admission of the patient to hospital. Put NA if this laboratory test is not available.
42	D-dimer (ng/mL)	Mandatory/ Personalised Information	Inform the D-dimer value as in the test made at the admission of the patient to hospital. Put NA if this laboratory test is not available.
43	Ferritin (ng/mL)	Mandatory/ Personalised Information	Inform the ferritin value as in the test made at the admission of the patient to hospital. Put NA if this laboratory test is not available.
44	Fibrinogen (g/L)	Personalised Information	Inform the fibrinogen value as in the test made at the admission of the patient to hospital. Put NA if this laboratory test is not available.
45	Lactate	Mandatory	Fill in this field if the lactate is: 1 – Arterial; 2 – Venous; 3 – Not applicable, if the test is not performed.
46	Lactate Value	Personalised Information	Inform the lactate value in the test made on the patient's admission to hospital. This item shall not be shown if the option “Not applicable” is selected.
47	Lactate Unit	Personalised Information	Inform the measuring unit as registered in the previous unit (mmol/L or mg/dL) used as part of the tests performed on the admission of the patient to hospital.
48	Lactate dehydrogenase (LDH) (U/L)	Mandatory/ Personalised Information	Inform the LDH value in the test made on the patient's admission to hospital. This item shall not be shown if the option “Not applicable” is selected.
49	NT-pro-BNP (pg/mL)	Personalised Information	Inform the NT-pro-BNP value in the test made on the patient's admission to hospital. Put NA if the test is not available.
50	CRP (mg/L)	Personalised Information	Inform the CRP value in the test made on the patient's admission to hospital. Put NA is the test is not available.
51	Procalcitonin (ng/ml)	Mandatory/ Personalised Information	Inform the Procalcitonin value in the test made on the patient's admission to hospital. Put NA if the test is not available.
52	Potassium (mmol)	Personalised Information	Inform the Potassium level value in the test made on the patient's admission to hospital. Put NA if the test is not available.
53	aPPT (seconds) / control	Personalised Information	Inform the aPTT value in the test made on the patient's admission to hospital. Put NA if the test is not available.
54	RNI	Personalised Information	Inform the RNI value in the test made on the patient's admission to hospital. Put NA if the test is not available.

55	Sodium (mmol)	Mandatory/ Personalised Information	Inform the Sodium level in the test made on the patient's admission to hospital. Put NA if the test is not available.
56	GOT/AST (U/L)	Mandatory/ Personalised Information	Inform the GOT/AST level in the test made on the patient's admission to hospital. Put NA if the test is not available.
57	GPT/ALT (U/L)	Mandatory/ Personalised Information	Inform the GOT/ALT level in the test made on the patient's admission to hospital. Put NA if the test is not available.
58	Troponine	Personalised Information	Inform the Troponin Value in the test made on the patient's admission to hospital. Put NA if the test is not available.
59	Urea (mg/dL)	Personalised Information	Inform the urea level in the test made on the patient's admission to hospital. Put NA if the test is not available.
60	pH	Personalised Information	Inform the pH as part of the arterial gasometry test, on the patient's admission to hospital. Put NA if the test is not available.
60.1	Arterial pCO_2	Personalised Information	Inform the pCO_2 value within the arterial gasometry test on the patient's admission to hospital. Put ND if the test is not available.
60.2	Arterial pO_2	Mandatory/ Personalised Information	Inform the pCO_2 value within the arterial gasometry test on the patient's admission to hospital. Put ND if the test is not available.
60.3	HCO_3^-	Personalised Information	Inform the HCO_3^- value within the arterial gasometry test on the patient's admission to hospital. Put ND if the test is not available.
60.4	FiO_2 (at the moment of collection of gasometrics)*	Mandatory/ Personalised Information	Inform the FiO_2 value at the moment of collection for the arterial gasometry test on the patient's admittance to hospital. Put NA if the test is not available. **This item of data is essential for the calculation of the ratio between PaO2/FiO2, meaning that it is very important to try to obtain this.**

* If the patient is not on mechanical ventilation, the use of the following estimates for FiO_2 should be filled in:

Device	Flow (L/min)	Approximate value of FiO2 to be used in the collection form
**No – regular room air**	0	0.21
**Nasal Cannula**	1	0.24
	2	0.28
	3	0.32
	4	0.36
	5	0.40
	6	0.44
**Simple Mask**	5	0.40
	6	0.50
	7	0.60
**Mask with Non-Reinhaling Reservoir**	6	0.60
	7	0.70
	8-9	0.80
	10-15	0.95

Electrocardiographic Findings – Admission			
**Nº**	**Item**	**Options for Answer**	**Guidance for Completion**
61	ECG carried out within the period of 24 hours after admission?	Mandatory	Reply with ‘yes’ or ‘no’
62	QT Interval (ms)	Mandatory/ Personalised Information	Inform the value of the QT interval in the ECG test.
62.1	QTC interval (ms) (note down if there is no measured QT registered on the medical form, only QTc)	Conditional/ Personalised Information	Inform the corrected QT interval (QTc) **only if there is no QT interval as measured and if the QTc has been recorded.**
63	Heart Frequency	Mandatory/ Personalised Information	Inform the heart frequency in the ECG test.
64	Rhythm	Mandatory/ Conditional	Select the cardiac rhythm as seen in the ECG test, among the following options: 1 – Fibrillation/Atrial Flutter; 2 – Pacemaker; 3 – Multifocal Atrial Rhythm; 4 - Sinusal; 5 – SV Tachycardia; 6 – Monomorphic V-tach; 7 – Polymorphic V-tach; 8- Other.
65	Alterations	Mandatory/ Conditional	Select the alteration(s) as present in the ECG test, among the following: 1 – Primary Alterations of Repolarisation; 2 – Blockage of the Right Branch; 3 – Blockage of the Left Branch; 4 – 1st Grade BAV; 5 – 2nd Grade BAV; 3 – BAVT; 4 – Upper Left Hemiblock; 5 – Pathological Q Wave; 6 – LV Overload with changes in ST-T; 7 – None of the above.

Radiological Findings – Admittance to Hospital			
**N°**	**Item**	**Options for Answer**	**Guidance for Completion**
66	Has the chest X-ray been performed within the period of 24 hours?	Mandatory	Fill in with ‘yes’ or ‘no’
66.1	Findings of the chest X-ray	Mandatory/ Conditional	If ‘yes’ was chosen in the previous item, then please select the option(s) that apply to the findings that apply to the chest X-ray (multiple answers are accepted):<br>1- Atelectasis; 2- Cavitation; 3- Pleural Thickening; 4- Diffuse Interstitial Infiltrate; 5- Focal Interstitial Infiltrate; 6- Opaqueness in bilateral glazed glass; 7- Opaqueness in unilateral glazed glass;<br>8- Opaqueness in central glazed glass; 9 – Opaqueness in peripheral glazed glass; 10 – Pneumothorax;<br>11- None of the above.

			**If the X-Ray was taken but no statement was drawn up, and neither is there a report on the interpretation of the test on the medical file:**<br>**- If a medical collector, then appraise the test and mark the result based on the analysis. Make a remark on the spreadsheet for the control of the centre. Include a remark on the control spreadsheet of the interpretation centre, by the collector.**<br>**- If a non-medical collector, leave this variable blank.**
66.2	Is the RX analysis statement made by a radiologist?	Mandatory/ Conditional	Answer with 'yes' or 'no'.
67	Has the chest tomography been carried out within the period of 24 hours after admission to hospital?	Mandatory	Answer with 'yes' or 'no'.<br>**If the patient came transferred from another service and has had a chest tomography at the service where he/she was first admitted to hospital, then this tomography can be considered. Include a remark on the centre control spreadsheet, informing that the tomography was not performed at the current hospital.**
67.1	Findings of the chest tomography	Mandatory/ Conditional	If 'yes' was selected in the previous item, then please select the option(s) most applicable to the findings of the chest tomography (multiple answers are acceptable):<br>1- Atelectasis; 2- Cavitation; 3-Consolidation; 4- Pleural Effusion; 5- Vascular Thickening; 6- Lump with Halo in Glazed Glass; 7- Standard in Unilateral Glazed Glass; 8- Standard in Bilateral Glazed Glass; 9 – Standard in Central Glazed Glass; 10 – Standard in Peripheral Glazed Glass; 11 – Paving in Mosaic; 12 – Reversed Halo Sign; 13 – None of the above
67.2	Has there been any attack upon > 25% of the parenchyma in chest tomography?	Mandatory/ Conditional	Please complete with 'yes', 'no', or 'no information and non-medical collector'.<br>**If there is no record on the medical file, mentioning the percentage of the pulmonary parenchyma affected, and the data collector is a doctor, then check to see if, through visual appraisal of the image, there is at least 25% of the pulmonary parenchyma affected. A remark must be included on the control spreadsheet, stating that this information was appraised by the data collector.**<br>**If there is no record of the percentage, and the data collector is not a doctor, then mark the option 'no information and non-medical collector'.**
67.3	Has the tomography analysis statement been drawn up by a radiologist doctor?	Mandatory/ Conditional	Answer with 'yes' or 'no'.
68	Has the echocardiogram been carried out within 24 hours after	Mandatory	Choose one of the options: 'yes' or 'no'.

	admission to hospital?		
68.1	LVEF (%)	Mandatory/ Personalised Information	If you have answered ‘yes’ to the previous item, then please inform the value of the ejection fraction (LVEF) on the echocardiogram.
68.2	Segment Alteration	Mandatory/ Conditional	If you have answered ‘yes’ to the ‘Echocardiogram’ item, then choose one of the options: either ‘yes’ or ‘no’.
68.3	Type of Segment Alteration	Mandatory/ Conditional	If you have answered ‘yes’ to the previous item, then select the type of segment change as present on the echocardiogram: 1 – Akinesia; 2 – Dyskinesia; 3 – Hypokinesia.

1. Hospitalization

**Clinical Evaluation – Hospitalization (clinical evaluation at D3 as from hospitalization)**<br>In case of discharge from hospital before D3, the form shall be kept blank and saved as complete			
**N°**	**Item**	**Options for Answer**	**Guidance for Completion**
69	Glasgow Coma Scale	Mandatory/ Personalised Information	Inform the Glasgow score of the patient at D3 after hospitalisation. Put NA if this information is not available.<br>**If there is a record on the file of an alert and lucid patient, or use of the abbreviations LOTE, BOTE or LOCV, then consider a Glasgow score of 15.**<br>**If the patient is under continuous sedation, then fill in with NA (not applicable).**
69.1	State/Level of Consciousness	Conditional	If the Glasgow Coma Scale at D3 is not completed or not available, then please choose the option applicable to the patient: 1 – Lucid and alert; 2 – Confused; 3 -Disoriented; 4 – Drowsy; 5 – In a state of torpor; 6 – Comatose.<br>**If the patient is under continuous sedation, then please mark ‘Comatose’.**
70	Systolic Blood Pressure	Mandatory/ Personalised Information	Inform the systolic blood pressure in mmHg at D3 after hospitalisation. Put NA if this information is not available.
70.1	Diastolic Blood Pressure	Personalised Information	Inform the diastolic blood pressure in mmHg at D3 after hospitalisation. Put NA if this information is not available.
71	If there is monitoring of IBP; mean arterial pressure (mmHg)	Personalised Information	If this is a patient with monitoring of invasive blood pressure, then please inform the mean arterial blood pressure. Put NA if the information is not available.
72	Heartbeat Frequency	Personalised Information	Inform the heartbeat frequency in beats per minute (bpm) at D3 after hospitalisation. Put NA if the information is not available.
73	Breathing Frequency	Mandatory/ Personalised Information	Inform the breathing frequency in Inc/min as at D3 after hospitalisation. Put NA if the information is not available.
74	Saturation of O_2	Mandatory/ Personalised Information	Inform the saturation of O_2 in % at D3 as from hospitalisation. Put NA if the information is not available.

75	Environmental Air	Mandatory	Select one of the options, ‘yes’ or ‘no’, according to the supply of O_2 when the saturation of the previous item is registered, as at D3 after hospitalisation.
75.1	Nasal Catheter (NC)	Mandatory/ Conditional	If the ‘no’ option is selected in the ‘Environmental Air’ item, then the option of oxygen supply through a NC shall become available. Select one of the options, ‘yes’ or ‘no’, based on the situation as at D3 after hospitalisation
75.2	Flow	Personalised Information	If the previous item has been answered with ‘yes’, please inform the flow (L/min) of O2 as offered by nasal catheter. Put NA if the information is not available.
75.3	Face Mask	Mandatory/ Conditional	If the ‘no’ option has been chosen in the Environmental Air’ and ‘Nasal Catheter’ items, then the option of oxygen supply by face mask (FM) shall then become available. Select one of the options, ‘yes’ or ‘no’, based on the situation at D3 after hospitalisation.
75.4	Flow	Personalised Information	If the previous item has been answered as ‘yes’, then please inform the flow (L/min) of O_2 as offered by a face mask, as at D3 after hospitalisation. Put NA if the information is not available.
75.5	Invasive Mechanical Ventilation	Mandatory/ Conditional	If the ‘no’ option has been chosen in the Environmental Air’, ‘Nasal Catheter’, and ‘Face Mask’ items, then the option of oxygen supply by mechanical ventilation (IMV) shall then become available Select one of the options, ‘yes’ or ‘no’, based on the situation at D3 after hospitalisation.
76	Is there a record of the SOFA score at D3?	Mandatory	Select one of the options: ‘yes’ or ‘no’, reflecting the situation at D3 after hospitalisation.
76.1	What is the value?	Mandatory/ Personalised Information	If the ‘yes’ option is selected in the previous item, then please inform the SOFA score as at D3 after hospitalisation.
77	Is there a record of the SAPS-3 score at D3?	Mandatory	Select one of the options: ‘yes’ or ‘no’, reflecting the situation at D3 after hospitalisation.
77.1	SAPS-3	Mandatory/ Personalised Information	If the ‘yes’ option has been chosen in the previous item, then please inform the SAPS-3 score as at D3 after hospitalisation. This item will be available if the ‘no’ option is selected for the ‘is there a record of the SOFA score’.

**Clinical Appraisal – Hospitalisation (clinical evaluation at D5 after hospitalisation)**<br>If the patient is discharged before D5, then leave the form blank and save it as complete			
**N°**	**Item**	**Options for Answer**	**Guidance for Completion**
78	Glasgow Coma Scale	Mandatory/ Personalised Information	Inform the Glasgow score of the patient at D3 after hospitalisation. Put NA if this information is not available.<br>**If there is a record on the file of an alert and lucid patient, or use of the abbreviations LOTE, BOTE or LOCV, then consider a Glasgow score of 15.**

			**If the patient is under continuous sedation, then fill in with NA (not applicable).**
78.1	State/Level of Consciousness	Conditional	If the Glasgow Coma Scale at D5 is not completed or not available, then please choose the option applicable to the patient: 1 – Lucid and alert; 2 – Confused; 3 -Disoriented; 4 – Drowsy; 5 – In a state of torpor; 6 – Comatose. **If the patient is under continuous sedation, then please mark 'Comatose'.**
79	Systolic Blood Pressure	Mandatory/ Personalised Information	Inform the systolic blood pressure in mmHg at D5 after hospitalisation. Put NA if this information is not available.
79.1	Diastolic Blood Pressure	Personalised Information	Inform the diastolic blood pressure in mmHg at D5 after hospitalisation. Put NA if this information is not available.
80	If monitoring of IBP, please inform average blood pressure (mmHg)	Personalised Information	If a patient with monitoring of invasive blood pressure, then inform the mean arterial pressure. Put ND if this information is not available.
81	Heartbeat Frequency	Mandatory / Personalised Information	Inform the heartbeat frequency in beats per minute (bpm) at D5 after hospitalisation. Put NA if the information is not available.
82	Breathing Frequency	Mandatory/ Personalised Information	Inform the breathing frequency in Inc/min as at D5 after hospitalisation. Put NA if the information is not available.
83	Saturation of O_2	Mandatory/ Personalised Information	Inform the level of O_2 saturation in % as at D5 after hospitalisation. Put NA if the information is not available.
84	Environmental Air	Mandatory	Select one of the options: 'yes' or 'no', as at D5 as from hospitalisation.
84.1	Catheter nasal	Mandatory/ Conditional	<u>If the 'no' option is selected for the 'Environmental Air' item, then the option of offering oxygen through a nasal catheter shall be made available.</u> Select one of the options: 'yes' or 'no', as at D5 after hospitalisation
84.2	Flow	Personalised Information	If the previous item has been answered as 'yes', then please inform the flow (L/min) of O_2 as offered by a nasal catheter, on D5 as from hospitalisation. Put NA if the information is not available.
84.3	Face Mask	Mandatory/ Conditional	<u>If the 'no' option is selected for the 'Environmental Air' and 'Nasal Catheter' items, then the option of offering oxygen through a face mask shall be made available.</u> Select one of the options: 'yes' or 'no', as at D5 after hospitalisation.
84.4	Flow	Personalised Information	If the 'yes' option is selected for the previous item, then please inform the flow (L/min) of O_2 as provided by a face mask, as at D5 after hospitalisation. Put NA if the information is not available.
84.5	Invasive mechanical ventilation	Mandatory/ Conditional	<u>If the 'no' option is selected for the 'Environmental Air', 'Nasal Catheter' and 'Face Mask' items, then the option of offering oxygen through mechanical ventilation (MV) shall be made available.</u> Select one of the options: 'yes' or 'no', as at D5 after hospitalisation.

85	Is there a record of the SOFA score at D5	Mandatory	Select one of the options: ‘yes’ or ‘no’, as at D5 as from hospitalisation.
85.1	What is the value described?	Mandatory/ Conditional	If the ‘yes’ option is selected in the previous item, please inform the SOFA score as at D5 after hospitalisation.
86	Is there a record of the SAPS-3 score at D5?	Mandatory	Select one of the options: ‘yes’ or ‘no’, as at D5 as from hospitalisation.
86.1	SAPS-3	Mandatory/ Conditional	Inform the SAPS-3 score at D5 of hospitalisation. The item shall be available should ‘no’ have been selected for the item ‘is there a description of the SOFA score’.

**Laboratory Findings – Hospitalization**			
**N°**	**Item**	**Options for Answer**	**Guidance for Completion**
**Please note down the minimum and maximum values of the following events, during hospitalisation**			
88	Haemoglobin (g/dL) – minimum value	Personalised Information	Inform the lowest absolute level of blood haemoglobin during the hospitalisation of the patient. Put NA if the test is not available.
	Haemoglobin Date (min)	Personalised Information	Inform the date of collection for the haemoglobin test with the lowest absolute value of haemoglobin in the CBC during the hospitalisation of the patient.*
89	Haemoglobin (g/dL) – maximum value	Personalised Information	Inform the highest absolute level of blood haemoglobin during the hospitalisation of the patient. Put NA if the test is not available.
	Haemoglobin Date (max)	Personalised Information	Inform the date of collection for the haemoglobin test with the highest absolute value of haemoglobin in the CBC during the hospitalisation of the patient.*
90	Leukocytes (cells/mm^3) – minimum value	Mandatory/ Personalised Information	Inform the lowest absolute value of leukocytes in the CBC during the hospitalisation of the patient. Put NA if the test is not available.
91	Neutrophils (cells/mm^3) – minimum value **(the same test as the lowest leukocyte count)**	Personalised Information	Inform the lowest absolute neutrophil count **in the same CBC where the lowest absolute neutrophil count was also observed** during the hospitalisation of the patient. Put NA if the test is not available.
	Leukocyte Date (min)	Personalised Information	Inform the date of collection of the test with the lowest absolute leukocyte count in the CBC during the hospitalisation of the patient.
92	Leukocytes (cells/mm^3) – maximum	Mandatory/ Personalised Information	Inform the highest absolute value of leukocytes in the CBC during the hospitalisation of the patient. Put NA if the test is not available.
93	Neutrophils (cells/mm^3) – maximum value **(the same test as the highest leukocyte count)**	Personalised Information	Inform the highest absolute neutrophil count **in the same CBC where the highest absolute neutrophil count was also observed** during the hospitalisation of the patient. Put NA if the test is not available.

	Leukocyte Date (max)	Personalised Information	Inform the date of collection of the test with the highest absolute leukocyte count in the CBC during the hospitalisation of the patient.*
94	Rods (cells/mm^3)– maximum value	Personalised Information	Inform the maximum absolute number of rods on the complete blood count (CBC) test, during the hospitalisation of the patient. Put NA if the test is not available. **Appraise the maximum level of rods, regardless of the other variables within the complete blood count test (CBC).**
	Rods Date (max)	Personalised Information	Inform the date of collection of the test with the highest absolute rod count in the CBC during the hospitalisation of the patient.*
95	Lymphocytes (cells/mm^3) – minimum value	Mandatory/ Personalised Information	Inform the minimum absolute number of rods on the complete blood count (CBC) test, during the hospitalisation of the patient. Put NA if the test is not available. **Appraise the minimum level of rods, regardless of the other variables within the complete blood count test (CBC).**
	Lymphocytes Date (min)	Personalised Information	Inform the date of collection of the test with the lowest absolute lymphocyte count in the CBC during the hospitalisation of the patient.*
96	Lymphocytes (cells/mm^3) – maximum value	Personalised Information	Inform the maximum absolute number of rods on the complete blood count (CBC) test, during the hospitalisation of the patient. Put NA if the test is not available. **Appraise the maximum level of rods, regardless of the other variables within the complete blood count test (CBC).**
	Lymphocytes Date (max)	Personalised Information	Inform the date of collection of the test with the highest absolute lymphocyte count in the CBC during the hospitalisation of the patient.*
97	Platelets (cells/mm^3) – minimum value	Mandatory/ Personalised Information	Inform the lowest platelet count on the CBC during the hospitalisation of the patient. Put NA if the test is not available.
	Platelets Date (min)	Personalised Information	Inform the date of collection of the test with the lowest absolute platelet count in the CBC during the hospitalisation of the patient.*
98	Platelets (cells/mm^3) – maximum value	Mandatory/ Personalised Information	Inform the highest platelet count on the CBC during the hospitalisation of the patient. Put NA if the test is not available.
	Platelets Date (min)	Personalised Information	Inform the date of collection of the test with the highest absolute platelet count in the CBC during the hospitalisation of the patient.*
99	Ionic Calcium (mg/dL) – minimum value	Personalised Information	Inform the minimum level of ionic calcium in this test, during the hospitalisation of the patient. Put NA if the test is not available.
	Calcium Date (min)	Personalised Information	Inform the date of collection of the test with the lowest level of ionic calcium during the hospitalisation of the patient.*
100	Ionic Calcium (mg/dL) – maximum value	Personalised Information	Inform the maximum level of ionic calcium in this test, during the hospitalisation of the patient. Put NA if the test is not available.
	Calcium Date (max)	Personalised Information	Inform the date of collection of the test with the highest level of ionic calcium during the hospitalisation of the patient.*
101	Potassium (mmoL/L) – minimum value	Personalised Information	Inform the lowest potassium level in this test, during the hospitalisation of the patient. Put NA if the test is not available.

	Potassium Date (min)	Personalised Information	Inform the collection date of the test with lowest potassium level, during the hospitalisation of the patient.*
102	Potassium (mmoL/L) – maximum value	Personalised Information	Inform the highest potassium level in this test, during the hospitalisation of the patient. Put NA if the test is not available.
	Potassium Date (max)	Personalised Information	Inform the collection date of the test with highest potassium level, during the hospitalisation of the patient.*
103	Sodium (mmoL/L) – minimum value	Personalised Information	Inform the lowest sodium level in this test, during the hospitalisation of the patient. Put NA if the test is not available.
	Sodium Date (min)	Personalised Information	Inform the collection date of the test with lowest sodium level, during the hospitalisation of the patient.*
104	Sodium (mmoL/L) – maximum value	Personalised Information	Inform the highest sodium level in this test, during the hospitalisation of the patient. Put NA if the test is not available.
	Sodium Date (min)	Personalised Information	Inform the collection date of the test with highest sodium level, during the hospitalisation of the patient.*
105	Troponine – minimum value	Personalised Information	Inform the lowest troponine level in this test, during the hospitalisation of the patient. Put NA if the test is not available.
	Troponine Date (min)	Personalised Information	Inform the collection date of the test with lowest troponine level, during the hospitalisation of the patient.*
106	Troponine – maximum value	Personalised Information	Inform the highest troponine level in this test, during the hospitalisation of the patient. Put NA if the test is not available.
	Troponine Date (max)	Personalised Information	Inform the collection date of the test with highest troponine level, during the hospitalisation of the patient.*
**Note down JUST the maximum value shown in the tests below, during the period of hospitalisation**			
107	Total Bilirubin (mg/dL) – maximum value	Mandatory/ Personalised Information	Inform the maximum total bilirubin count in the test, during the hospitalisation of the patient. Put NA if the test is not available.
107.1	Direct bilirubin (mg/dL) – maximum value (from the same test as total bilirubin)	Personalised Information	Inform the maximum total bilirubin count in the test, during the hospitalisation of the patient. Put NA if the test is not available. **Use the same test where the maximum value of total bilirubin was obtained.**
	Bilirubin Date (max)	Personalised Information	Inform the date of collection of the test with the highest bilirubin count during the hospitalisation of the patient.*
108	BNP – maximum value	Mandatory/ Personalised Information	Inform the highest BNP level in the test, during the hospitalisation of the patient. Put NA if the test is not available.
	BNP (max)	Personalised Information	Inform the date of collection of the test with the highest BNP count during the hospitalisation of the patient.*
108.1	NT-pro-BNP – maximum value	Mandatory/ Personalised Information	Inform the highest NT-pro-BNP level in the test, during the hospitalisation of the patient. Put NA if the test is not available.

	NT-pro-BNP Date (max)	Personalised Information	Inform the date of collection of the test with the highest NT-pro-BNP count during the hospitalisation of the patient.*
109	Creatinin (mg/dL) – maximum value	Mandatory/ Personalised Information	Inform the highest Creatinin level in the test, during the hospitalisation of the patient. Put NA if the test is not available.
	Creatinin Date (max)	Personalised Information	Inform the date of collection of the test with the highest Creatinin count during the hospitalisation of the patient.*
110	Creatinophosphokinase (CPK - U/L) – maximum value	Mandatory/ Personalised Information	Inform the highest CPK value in the test on admission of the patient to hospital. Put ND if the test is not available.
	CPK Date (max)	Personalised Information	Inform the date of collection of the test with the highest CPK level, during the hospitalisation of the patient.*
111	D-dimer (ng/mL) – maximum value	Mandatory/ Personalised Information	Inform the highest D-dimer value in the test on hospitalisation of the patient. Put ND if the test is not available.
	D-dimer data (max)	Personalised Information	Inform the date of collection of the test with the highest D-dimer level, during the hospitalisation of the patient.*
112	Ferritin (ng/mL) – maximum value	Mandatory/ Personalised Information	Inform the highest Ferritin level in the test on hospitalisation of the patient. Put ND if the test is not available.
	Ferritin Date (max)	Personalised Information	Inform the date of collection of the test with the highest Ferritin level, during the hospitalisation of the patient.*
113	Lactate (mmol/L) – maximum value	Mandatory/ Personalised Information	Inform the highest Lactate level in the test on hospitalisation of the patient. Put ND if the test is not available.
	Lactate Date (max)	Personalised Information	Inform the date of collection of the test with the highest Lactate level, during the hospitalisation of the patient.*
113.1	Lactate (mmol/L) – medium	Mandatory	Inform if the lactate with highest level is: 1 – Arterial; 2 – Venous; 3 – Not applicable, if the test is not performed
113.2	Lactate (mmol/L) – unit	Mandatory	Inform if the collected lactate at maximum value is in the unit: 1 – mg/dL; 2 – mmol/L; 3 – Not applicable, if the test is not performed.
114	Lactate dehydrogenase (LDH) (U/L) – maximum value	Mandatory/ Personalised Information	Inform the highest LDH Reading in the tests, during the hospitalisation of the patient. Use NA if this test is not available.
	LDH Date (max)	Personalised Information	Inform the date of collection of the test with the highest LDH level, during the hospitalisation of the patient.*
115	CRP (mg/L) – maximum value	Personalised Information	Inform the highest CRP Reading in the tests, during the hospitalisation of the patient. Use NA if this test is not available.
	CRP Date (max)	Personalised Information	Inform the date of collection of the test with the highest CRP level, during the hospitalisation of the patient.*
116	Procalcitonin – maximum value	Mandatory/ Personalised Information	Inform the procalcitonin value on the test at hospitalisation of the patient. Use NA if the test is not available.
	Procalcitonin Date (max)	Personalised Information	Inform the date of collection of the test with the highest Procalcitonin level, during the hospitalisation of the patient.*

117	aPPT (seconds) / control – maximum value	Mandatory/ Personalised Information	Inform the highest aPPT level during test during the hospitalisation of the patient. Complete with NA if the test is not available.
	Data aPPT (max)	Personalised Information	Inform the date of collection of the test with the highest aPPT level, during the hospitalisation of the patient.*
118	RNI – maximum value	Mandatory/ Personalised Information	Inform the highest RNI level during test during the hospitalisation of the patient. Complete with NA if the test is not available.
	Data RNI (max)	Personalised Information	Inform the date of collection of the test with the highest RNI level, during the hospitalisation of the patient.*
119	GOT/AST (U/L) – maximum value	Mandatory/ Personalised Information	Inform the highest GOT/AST level during test during the hospitalisation of the patient. Complete with NA if the test is not available.
	GOT/AST Date (max)	Personalised Information	Inform the date of collection of the test with the highest GOT/AST level, during the hospitalisation of the patient.*
120	GPT/ALT (U/L) – maximum value	Mandatory/ Personalised Information	Inform the highest GPT/ALT level during test during the hospitalisation of the patient. Complete with NA if the test is not available.
	Data GPT/ALT (max)	Personalised Information	Inform the date of collection of the test with the highest GPT/ALT level, during the hospitalisation of the patient.*
121	Urea (mg/dL) – maximum value	Personalised Information	Inform the highest urea level in a test during the hospitalisation of the patient. Complete with NA if the test is not available.
	Urea Date (max)	Personalised Information	Inform the date of collection of the test with the highest urea, during the hospitalisation of the patient.*
**Note down ONLY the lowest value of the following tests during hospitalisation**			
122	Albumin (g/dL) – minimum value	Personalised Information	Inform the lowest albumin level in a test during the hospitalisation of the patient. Complete with NA if the test is not available.
	Albumin Date (min)	Personalised Information	Inform the date of collection of the test with the lowest albumin level, during the hospitalisation of the patient.*
123	pO_2 arterial – minimum value	Personalised Information	Inform the lowest pO_2 level in arterial gasometry test during the hospitalisation of the patient. Use NA if the test is not available.
	pO_2 Date (min)	Personalised Information	Inform the date of collection of the test with the lowest pO_2 level, during the hospitalisation of the patient.*
123.1	FiO_2 related to pO_2 – minimum value	Personalised Information	Inform the minimum value of FiO_2 at the moment of collection of the arterial gasometry test with the lowest pO_2 during the hospitalisation of the patient. Use NA if the test is not available.
123.2	pH – minimum value	Mandatory/ Personalised Information	Inform the lowest pH level in arterial gasometry test during the hospitalisation of the patient. Use NA if the test is not available.
123.3	Arterial pCO_2	Personalised Information	Inform the pCO_2 – **As a reference, use the same test in which the minimum pH value was obtained.** Use NA if the test is not available.
123.4	HCO_3 – minimum value	Personalised Information	Inform the HCO_3- level. **As a reference, use the same test where the minimum pH value was obtained.** Use NA if the test is not available.

	Gasometry Date	Personalised information	Date of collection of gasometry with lowest pH*
124	Is there any laboratory confirmation of coinfection with dengue?	Mandatory	Answer with ‘yes’ or ‘no’.
124.1	Describe the method of confirmation	Conditional	If ‘yes’ was selected in the previous item, then please select the method of confirmation of infection with dengue: 1 – rapid testing and 2 – serology.

* **If the same value is obtained on two different dates, then the first date shall be considered,** with the exception of tests on admittance to hospital, which are already collected.

For those tests which require a maximum and minimum value, if the patient has only one available test, then the result shall be repeated as maximum and minimum.

**Radiological Findings – Hospitalisation (First test after the first 24 hours of hospitalisation)**			
**N°**	**Item**	**Options for Answer**	**Guidance for Completion**
125	Has the chest X-ray been performed after 24 hours of hospitalisation?	Mandatory	Answer with ‘yes’ or ‘no’.
125.1	Findings of chest X-ray	Mandatory/ Conditional	If ‘yes’ has been selected in the previous item, please select the option(s) that correspond to the findings in the chest X-ray. (Multiple answers are accepted):<br>1- Atelectasis; 2- Cavitation; 3- Pleural Thickening; 4 – Diffuse Interstitial Infiltrate; 5 – Focal Interstitial Infiltrate; 6 – Opaqueness in Bilateral Glazed Glass; 7 – Opaqueness in Unilateral Glazed Glass; 8 – Opacity in Central Glazed Glass; 9 – Opaqueness in Peripheral Glazed Glass; 10 – Pneumothorax; 11 – None of the Above.<br>**If there has been an X-ray but no statement or report on the interpretation of the tests in a medical record:**<br>**- If the data collector is a doctor, then assess the test and mark the result based on the analysis. Include a remark on the control spreadsheet of the interpretation centre, by the data collector.**<br>**- If the data collector is not a doctor, then leave this variable blank.**
125.2	Has the interpretative statement of the X-Ray been drawn up by a radiologist doctor?	Mandatory/ Conditional	Answer with ‘yes’ or ‘no’.
126	Was the chest TC been carried out 24	Mandatory	Answer with ‘yes’ or ‘no’.

	hours after hospitalisation?		
126.1	Findings in the chest tomography	Mandatory/ Conditional	If ‘yes’ has been selected in the previous item, please select the option(s) that correspond to the findings in the chest tomography. (Multiple answers are accepted):<br>1- Atelectasis; 2- Cavitation; 3- Consolidation; 4 – Pleural Effusion; 5 – Vascular Thickening; 6 – Lump with Halo in Glazed Glass; 7 – Standard in Unilateral Glazed Glass; 8 – Standard in Bilateral Glazed Glass; 9 – Standard in Central Glazed Glass; 10 – Standard in Peripheral Glazed Glass; 11 – Paving in Mosaic; 12 – Reversed Halo Sign; 13 - None of the Above.
126.2	Has more than 25% of the pulmonary parenchyma been affected as shown in the chest TC?	Mandatory/ Conditional	If ‘yes’ was selected in ‘Chest Tomography Carried Out?’ then complete with either ‘yes’, ‘no’, or ‘No Information, Date Collector Not a Doctor’.<br>**If there is no records of the percentage of pulmonary parenchyma affected, on the medical files, and the data collector is a doctor, then check, through the visual appraisal of the image, to see if there has been 25% of pulmonary parenchyma affected. Make a remark on the control spreadsheet, stating that this information was assessed by the data collector. If the percentage has not been recorded and the data collector is not a doctor, then mark the option ‘No information, Data Collector Not a Doctor’.**
126.3	Has the analysis statement of the TC been drawn up by a radiologist doctor?	Mandatory/ Conditional	If ‘yes’ was selected in ‘Chest Tomography Carried Out?’ then answer with ‘yes’ or ‘no’.
127	Has an echocardiogram been performed after 24 hours after hospitalisation?	Mandatory	Answer with ‘yes’, ‘no’ or ‘no information, data collector not a doctor’.
127.1	Ejection Fraction (%)	Mandatory/ Personalised Information	If the previous item is answered with ‘yes’, then please inform the ejection fraction value (LVEF) on the echocardiogram.
127.2	Segment Alteration	Mandatory/ Conditional	If the “Echocardiogram” item has been answered ‘yes’, then select either ‘yes’ or ‘no’
127.3	Type of Alteration	Mandatory/ Conditional	If the previous item has been answered with ‘yes’, then select the type of segment alteration that is present in the echocardiogram: 1 – Akinesia; 2 – Dyskinesia; 3 – Hypokinesia.

**Therapeutic Intervention**			
**N°**	**Item**	**Options for Answer**	**Guidance for Completion**

128	Therapies used during Hospitalisation	Mandatory	Select the option(s) referring to the therapy used during the whole period of hospitalisation of the patient (multiple answers are accepted): 1 – Antiarrhythmics; 2 – Antibiotics **(except Azithromycin and Clarithromycin)**; 3 – Anticoagulants; 4 – Antifungal Drugs; 5 – Non-steroidal Anti-Inflammatory Drugs (NSAIDs); 6 – Corticotherapy; 7 – Statins; 8 – Oseltamivir; 9 – None of the Above.
128.1	Which Anticoagulant?	Mandatory/ Conditional	If the ‘Anticoagulant’ option has been chosen in item 128, then select the item that refers to what anticoagulant was used: 1 – Unfractionated Heparin; 2 – Low Molecular Weight Heparin; 3 – Fondaparinux; 4 – Warfarin; 5 – Others.
128.2	Unfractionated Heparin Dose*	Mandatory/ Conditional	If the option ‘Unfractionated Heparin’ is selected in item 128.1, please select the option referring to the dose of anticoagulants: 1 – Prophylactic; 2 – Therapeutic
128.3	Start of Treatment with Unfractionated Heparin	Mandatory/ Personalised Information	If the option ‘Unfractionated Heparin’ is selected in item 128.1, please inform the date when the medication started to be used, in DD/MM/YYYY format.
128.4	End of Treatment with Unfractionated Heparin	Mandatory/ Personalised Information	If the option ‘Unfractionated Heparin’ is selected in item 128.1, please inform the date when the medication stopped being used, in DD/MM/YYYY format.
128.5	Dose of Low Molecular Weight Heparin [α]	Mandatory/ Conditional	If the option ‘Low Molecular Weight Heparin’ is selected in item 128.1, please select the option referring to the dose of anticoagulants: 1 – Prophylactic; 2 – Therapeutic
128.6	Start of Treatment with Low Molecular Weight Heparin	Mandatory/ Personalised Information	If the option ‘Low Molecular Weight Heparin’ is selected in item 128.1, please inform the date when the medication started to be used, in DD/MM/YYYY format.
128.7	End of Treatment with Low Molecular Weight Heparin	Mandatory/ Personalised Information	If the option ‘Low Molecular Weight Heparin’ is selected in item 128.1, please inform the date when the medication stopped being used, in DD/MM/YYYY format.
128.8	Dose of Fondaparinux[ƚ]	Mandatory/ Conditional	If the option ‘Fondparinux’ is selected in item 128.1, please select the option referring to the dose of anticoagulants: 1 – Prophylactic; 2 – Therapeutic
128.9	Start of Treatment with Fondparinux	Mandatory/ Personalised Information	If the option ‘Fondparinux’ is selected in item 128.1, please inform the date when the medication started to be used, in DD/MM/YYYY format.
128.10	End of Treatment with Fondparinux	Mandatory/ Personalised Information	If the option ‘Fondparinux’ is selected in item 128.1, please inform the date when the medication stopped being used, in DD/MM/YYYY format.
128.11	Start of Treatment with Warfarin	Mandatory/ Personalised Information	If the option ‘Warfarin’ is selected in item 128.1, please inform the date when the medication started to be used, in DD/MM/YYYY format.
128.12	End of Treatment with Warfarin	Mandatory/ Personalised Information	If the option ‘Warfarin’ is selected in item 128.1, please inform the date when the medication stopped being used, in DD/MM/YYYY format.
128.13	What other anticoagulant?	Conditional/ Personalised Information	If the option ‘Others’ is selected in item 128.1, please inform which other anticoagulant is being used.

128.14	Start of Treatment with Other Anticoagulant	Mandatory/ Personalised Information	If the option ‘Other Anticoagulant’ is selected in item 128.1, please inform the date when the medication started to be used, in DD/MM/YYYY format.
128.15	End of Treatment with Other Anticoagulant	Mandatory/ Personalised Information	If the option ‘Other Anticoagulant’ is selected in item 128.1, please inform the date when the medication stopped being used, in DD/MM/YYYY format.
128.16	Which Corticoid?	Mandatory/ Conditional	If the option ‘Corticoid’ is selected in item 128, then please mark if the corticoid used was: 1 – Dexamethasone, 2 – Another Corticoid.
128.17	Dose of Dexamethasone?	Mandatory/ Conditional	If the option ‘Dexamethasone’ is selected inn item 128.16, then mark if the dose was: 1 – 6 mg/day, 2 – Another dose.
128.18	Another dose of dexamethasone. What dose? (mg)	Conditional/ Personalised Information	If ‘Another Dose’ is selected in item 128.17, then inform the dose of dexamethasone in milligrams (numbers only)
128.19	Start of Treatment with Corticoid	Conditional/ Personalised Information	If the option ‘Corticoid’ is selected in item 128, please inform the date when the medication started to be used, in DD/MM/YYYY format.
128.20	End of Treatment with Corticoid	Conditional/ Personalised Information	If the option ‘Corticoid’ is selected in item 128, please inform the date when the medication stopped being used, in DD/MM/YYYY format.
129	Therapy Introduced Specifically for Covid-19	Mandatory	Select the option(s) referring to the therapies as used during the whole of the hospitalisation period (multiple answers are accepted): 1 – Azithromycin; 2 –Clarithromycin; 3-Chloroquine; 4 – Favipiravir; 5 – Hydroxychloroquine; 6 – Interferon; 7 – Immunoglobulin; 8 – Convalescent Plasma; 9 – Remdesivir; 10 – Ritonavir/Lopinavir; 11 – Sarilumab; 12 – Tocilizumab; 13 – Umifenovir (Arbidol); 14 – Other (Which?) 15 – None.
129.1	Dose of Chloroquine Used	Mandatory/ Conditional	If the option ‘Chloroquine’ has been selected in the previous item, please select the dose used: 1 – 450mg 12/12h on 1st day + 450mg 24/24h; 2 – 500mg 12/12h on the 1st day + 250mg 12/12h; 3 – Another dose. Which one?
129.2	Usage Time (in days)	Mandatory/ Personalised Information	If the option ‘Chloroquine’ has been selected in item 129, then please state for how long chloroquine was used in the patient (in days)
129.3	Describe Other Dose Used	Mandatory/ Personalised information	If ‘Another Dose’ is selected in the item ‘Dose of Chloroquine Used’, describe the dose used.
129.4	Were there any Adverse Side Effects associated with the use of Chloroquine or Hydrochloroquine?	Mandatory/ Conditional	Inform ‘yes’ or ‘no’, in relation to adverse side effects linked to the use of chloroquine or hydrochloroquine.
129.5	Adverse Effects Associated to the Use of Chloroquine or Hydrochloroquine	Mandatory/ Conditional	Mention what adverse side effects were shown by the patient: 1. Widening of the QT Interval; 2. Non-ischaemic cardiac dysfunction; 3. Fibrillation or Atrial Flutter; 4. Myopathy; 5. Neuropathy; 6. Gastrointestinal Symptoms; 7. Supraventricular Tachycardia (except FA/Flutter); 8. Monomorphic Ventricular Tachycardia; 9. Polymorphic Ventricular Tachycardia; 10. Others.

129.6	Other Adverse Effects Reported?	Mandatory/ Personalised Information	If the option 'others' is selected in the previous item, then please describe other adverse effects that are associated to the use of Chloroquine and Hydrochloroquine.
129.7	Has the use of Chloroquine and Hydrochloroquine been Suspended Thanks to Adverse Effects?	Mandatory	Answer with 'yes' or 'no'.
129.8	Dose of Hydroxychloroquine Used	Mandatory/ Conditional	If the option 'Hydroxychloroquine' has been selected under 'Therapy Introduced Specifically for COVID-19', then please select the dose used: 1 - 400mg 12/12h on the 1st day + 200mg 8/8h; 2 - 400mg 12/12h on the 1st day + 200mg de 12/12h; 3 – 400mg 24/24h; 4 – 200mg 8/8h; 5 – Another Dose. Which one?
129.9	Describe another dose used	Mandatory/ Personalised Information	If 'Another Dose' has been selected in the previous item, please describe the dose used.
129.10	Days of Use	Mandatory/ Personalised Information	Describe the time over which Hydrochloroquine has been used with the patient (in days)
129.11	Describe Other Therapy Used	Mandatory/ Personalised Information	If the option 'Other – which one?' has been selected under 'Therapy Introduced Specifically for COVID-19", please describe the other therapy used.
130	Support Care	Mandatory	Select the option(s) related to support care as provided during the whole period of hospitalisation: 1 – Vasoactive Amines; 2 – ECMO; 3 – Prone position; 4 – Volemic Resuscitation; 5 – Non-Invasive Mechanical Ventilation; 6 – None of these
131	Definition of Palliative Care for the Patient	Mandatory	Answer with 'yes' or 'no'.

*** Dose of unfractionated heparin (UFH):** Doses considered **prophylactic** are UFH 5,000 UI applied subcutaneously (SC) every 12 hours (12/12h) or every 8 hours (8/8h). The **therapeutic** dose is normally given based on the weight of the patient, in a continuous infusion pump, with monitoring of aPPT. In exceptional cases, there are therapeutic doses at 320UI/kg of attack weight and 250UI/kg of weight 12/12h SC, without any monitoring of aPPT.

ᾳ **Dose of low molecular weight heparin:** Examples of **prophylactic** doses: enoxaparin 40mg SC 24/24h or dalteparin 5000UI 24/24h. Examples of **therapeutic** doses include: enoxaparin 1mg/Kg of body weight SC 12/12h or 1.5mg/Kg of body weight, every 24 hours; dalteparin 200UI/Kg SC every 24 hours. The therapeutic dose could be adjusted if there is kidney failure.

ⱡ **Dose of fondaparinux:** The prophylactic dose of fondaparinux is 2.5 mg SC every 24 hours. The therapeutic dose depends on weight, being set at 5mg if <50Kg, 7.5mg if 50-100Kg, and 10mg if >100Kg, SC, every 24 hours.

2. Discharge or In-hospital Mortality

Outcomes			
	**Item**	**Options for Answer**	**Guidance for Completion**
132	Date of Hospital Discharge / Death / Transfer	Mandatory	Inform the date of hospital discharge or death, using the DD/MM/YYYY format.
132.1	Was there a transfer to another institution?	Mandatory	Answer ‘yes’ or ‘no’, with regard to transfer to another institution.
132.2	What Institution?	Mandatory/ Conditional	If the answer to the previous item was ‘yes’, then describe the institution of transfer.
133	Was there admission to the Intensive Care Unit?	Mandatory	Answer ‘yes’ or ‘no’, with regard to whether the patient was admitted to intensive care.
133.1	Date of Admission to Intensive Care (ICU)	Mandatory/ Personalised Information	If the answer to the previous item was ‘yes’, then please inform the date of admission of the patient to intensive care treatment (ICU), using the DD/MM/YYYY format.
133.2	Date of Discharge from Intensive Care (ICU)	Mandatory/ Personalised Information	If the answer to item 142 is ‘yes’, then please supply the date when the patient was discharged from the intensive care unit (ICU), using the DD/MM/YYYY format. In the case of a death in intensive care, the date of death should be informed.
134	Was there any need for mechanical ventilation?*	Mandatory	Answer ‘yes’ or ‘no’, with regard to the need for mechanical ventilation
134.1	Days of mechanical ventilation	Mandatory/ Personalised Information	If the answer to the previous item is ‘yes’, then please inform the number of days when the patient remained under mechanical ventilation.
134.2	Was there any failure of extubation [α]	Mandatory	Answer ‘yes’ or ‘no’, in relation to failure of extubation.
135	Was there any need for replacement kidney therapy (dialysis)?[ǂ]	Mandatory	Answer ‘yes’ or ‘no’, with regard to the need for dialysis during the hospitalisation period.
136	Intercurrences during hospitalisation?[Ŧ]	Mandatory	Select the option(s) that correspond to the support care provided during the whole hospitalisation period (multiple answers are accepted): 1. Septic Shock; 2. Widespread Intravascular Coagulation; 3. Acute IC (new case, or decompensated chronic case); 4. Hospital Infection; 5. Heart Attack; 6. Myocarditis; 7. Haemorrhage; 8. Hyperglycaemia; 9. Adult Breathing Distress Syndrome; 10. Vascular Thrombosis; 11. Other; 12. None
136.1	Where is the Haemorrhage?	Mandatory/ Conditional	If ‘Haemorrhage’ was selected as part of ‘Intercurrences during Hospitalisation’, then please inform the site of the haemorrhage.
136.2	How serious is the haemorrhage?	Mandatory/ Conditional	If ‘Haemorrhage’ was selected as part of ‘Intercurrences during hospitalisation’, then choose one of the following alternatives: 1. Serious; 2. Not serious, but clinically relevant; 3. Not serious.[Ŧ]

136.3	What kind of thromboembolic event?	Mandatory/ Conditional	If ‘Vascular Thrombosis’ was selected under ‘Intercurrences During Hospitalisation’, then select one option corresponding to the type of thromboembolic event: 1 – DVT; 2 – TEP; 3 – Arterial Thrombosis.
136.4	Any other complications?	Mandatory/ Conditional	If ‘Other’ was selected under ‘Intercurrences During Hospitalisation’, then please select the other complication that was shown.
137	Was there any kind of worsening to heart and breathing stoppage?[€]	Mandatory	Answer with ‘yes’ or ‘no’.
137.1	Was any cardiovascular resuscitation performed?	Mandatory/ Conditional	Select the appropriate option: 1. Yes; 2. No; 3. Not described.
138	Death	Mandatory	Answer with ‘yes’ or ‘no’.

* **Need for mechanical ventilation:** Annotation on the medical record, confirming the need for mechanical ventilation

[α] **Fault of extubation:** Record on the medical file, confirming fault of extubation.

[ƚ] **Need for replacement kidney therapy:** An annotation on the medical records mentioning dialysis during hospitalisation that was started during hospitalisation (meaning that prior dialysis are excluded).

[Ŧ]Consider the following definition for intercurrences during hospitalisation:

1. **Septic Shock:** Annotation on the medical file mentioning septic shock; or, in the case of patients with evidence of infectious process: presence of shock; use of amines and lactate persistently over 2 mmol/L (18 mg/dL), despite appropriate volemic resuscitation
2. **Widespread Intravascular Coagulation:** Annotation on medical files or score as proposed by the *International Society on Thrombosis and Haemostasis* of ≥5, with this score being calculated automatically based on the information supplied on the form, taking into account factors such as platelet count, D-dimer, coagulogram and fibrinogen.
3. **Acute heart failure:** A new, or a decompensated chronic case of heart failure, regardless of whether the ejection fraction is preserved or reduced.
4. **Hospital Infection:** Record of a bacterial infectious process in any site, or at an undetermined site, diagnosed 48 hours after admission to hospital.
5. **Heart Attack (Myocardial Infarction)**: Medical files recording an acute myocardial infarction of any type.
6. **Myocarditis:** Diagnosis of myocarditis, duly recorded on the medical file.
7. **Haemorrhage:** Medical file mentioning hemorrhagic complication(s) which could be considered as (Variable 146.2):

- **Serious Haemorrhage:** Clinically evident bleeding leading to any of the following situations: death; involvement of a key anatomic site (intracranial; spinal; pericardial; articular; retroperitoneal; or intramuscular, with compartment syndrome); fall of at least 2 g/dL in the concentration of haemoglobin; shock; transfusion of at least 2 units of entire blood or RBC concentrate; or permanent invalidity.
- **Haemorrhage not serious, but clinically relevant:** Presence of evident bleeding that does not meet the criteria for defining serious bleeding, but which warranted medical intervention, temporary interruption of treatment, or that generated any kind of pain.
- **Haemorrhage not serious:** Do not fall under any of the previous criteria.

8. **Hyperglycaemia:** Blood sugar levels of over 180mg/dL, even for capillary blood, duly registered on the medical file.
9. **Adult Breathing Distress Syndrome:** Medical file mentioning adult breathing distress syndrome, ARDS, SDRA; or a diagnosis of disproportional hypoxaemia, registered on the medical file, through arterial gasometry with $pO_2/FiO_2<200$ at any moment; or through manoeuvres of alveolar recruitment.
10. **Vascular Thrombosis:** Diagnosis of arterial thrombosis registered on a medical file; deep venous thrombosis confirmed by imaging test (duplex scan or compression ultrasound); and/or pulmonary embolus by imaging test (angiotomography, scintillography; or, if there is haemodynamic instability and in the absence of confirmation through previous testing, changes that would suggest acute overload of the right ventricle, in an echocardiogram or bedside ultrasound).

[€]**Heart and respiratory stoppage:** Registered in a medical file, regardless of the rhythm of stoppage.

**Electrocardiographic Outcomes**			
**N°**	**Item**	**Options for Answer**	**Guidance for Completion**
139	Was there any electrocardiographic change during hospitalisation?*	Mandatory	Answer with ‘yes’ or ‘no’.
139.1	Changes in rhythm?	Mandatory/ Conditional	If ‘yes’ was selected in the previous item, then please select the options referring to the changes in the electrocardiographic rhythm present during the hospitalisation of the patient, including: 1. Fibrillation/Atrial flutter; 2. Pacemaker; 3. Multifocal atrial rhythm; 4. Supraventricular Tachycardia; 5. Monomorphic Ventricular Tachycardia; 6. Polymorphic Ventricular Tachycardia; 7. Supraventricular Extrasystole; 8 – Ventricular Extrasystole; 9. No change of rhythm.

139.2	Other electrocardiographic changes?	Mandatory/ Conditional	If ‘yes’ was selected in the item ‘Has there been any electrocardiographic change during hospitalisation’, select the options that show the electrocardiographic changes that were present during hospitalisation, including: 1. Widening of the QT interval; 2. Primary changes to repolarisation; 3. Blockage of the right branch; 4. Blockage of the left branch; 5. 1st grade BAV;6. 2nd grade BAV; 7. BAVT; 8. Anterior left hemiblock; 9. Pathological Q Waves; 10. Overload of LV with changes to ST-T; 11. Other; 12. No changes.
139.3	Describe any other electrocardiographic alteration	Mandatory/ Personalised Information	If ‘Other’ is selected in the previous item, please describe the electrocardiographic changes found.
