# Supplementary material for: Clinical characteristics and outcomes of COVID-19 patients with preexisting dementia: a large multicenter propensity-matched Brazilian cohort study
Source: BMC Geriatr. 2024 Jan 5;24:25. doi: 10.1186/s12877-023-04494-w (PMC10770897; doi:10.1186/s12877-023-04494-w)
Supplement: Supplementary file 2 — Additional file 2: Table S1. Laboratory exams among the included COVID-19 patients with dementia and matched controls without dementia. [file 12877_2023_4494_MOESM2_ESM.pdf]

Table S1. Laboratory exams among the included COVID-19 patients with dementia and matched controls without dementia

Variables	Dementia N= 405[1]	Control patients N= 1,151[1]	p-value[2]
Hemogram			
Haemoglobin (g/L)	12.1 (10.9, 13.3)	12.4 (11.2, 13.6)	0.006
Leukocytes count (cels/mm^3)	7,380.0 (5,397.2, 10,762.5)	7,741.0 (5,557.5, 10,990.0)	0.711
Neutrophiles (cels/mm^3)	5,749.0 (3,770.0, 8,700.0)	6,100.0 (3,979.4, 8,940.0)	0.355
Lymphocytes (cels/mm^3)	880.0 (607.0, 1,302.0)	860.0 (610.0, 1,227.0)	0.563
Platelet count (cels/mm^3)	189,000.0 (138,000.0, 243,000.0)	194,000.0 (148,000.0, 258,000.0)	0.078
Other laboratory results			
TGO/AST (U/L)	39.0 (28.0, 59.8)	39.0 (28.0, 57.0)	0.959
TGP/ALT (U/L)	25.0 (17.0, 41.0)	26.0 (18.0, 39.2)	0.810
pH	7.4 (7.4, 7.5)	7.4 (7.4, 7.5)	0.737
PO_2	74.9 (61.0, 95.3)	74.2 (62.3, 97.2)	0.794
PCO_2	35.0 (30.7, 39.1)	35.0 (31.0, 40.0)	0.384
Bicarbonate	23.3 (20.9, 26.0)	23.0 (20.9, 25.9)	0.463
Sodium (mmol/L)	139.0 (137.0, 144.0)	137.0 (134.0, 141.0)	<0.001
Creatinine (mg/dL)	1.0 (0.8, 1.6)	1.1 (0.8, 1.5)	0.792
INR	1.1 (1.0, 1.2)	1.1 (1.0, 1.2)	0.215
Lactate	1.5 (1.1, 2.0)	1.6 (1.1, 2.1)	0.297
Urea (mg/dL)	55.0 (39.0, 92.4)	53.0 (37.3, 80.8)	0.044

[1]Median (IQR); n (%); [2]Wilcoxon rank sum test; Fisher's exact test. ALT: alanine aminotransferase; AST: aspartate transaminase; INR: international normalised ratio; PaO_2: partial pressure of oxygen; $PaCO_2$: partial pressure of carbon dioxide; TGO/AST: aspartate aminotransferase; TGP/ALT: Alanine aminotransferase.
